# Predicting adenine base editing efficiencies in different cellular contexts by deep learning

**DOI:** 10.1186/s13059-025-03586-7

**Published:** 2025-05-08

**Authors:** Lucas Kissling, Amina Mollaysa, Sharan Janjuha, Nicolas Mathis, Kim F. Marquart, Yanik Weber, Woohyun J. Moon, Paulo J. C. Lin, Steven H. Y. Fan, Hiromi Muramatsu, Máté Vadovics, Ahmed Allam, Norbert Pardi, Ying K. Tam, Michael Krauthammer, Gerald Schwank

**Affiliations:** 1 Institute of Pharmacology and Toxicology, University of Zurich, Zurich, Switzerland; 2 Department of Quantitative Biomedicine, University of Zurich, Zurich, Switzerland; 3 Institute of Molecular Health Sciences, ETH Zurich, Zurich, Switzerland; 4 Department of Microbiology, Perelman School of Medicine, University of Pennsylvania, Philadelphia, PA USA; 5 Acuitas Therapeutics Inc., Vancouver, BC Canada

**Keywords:** Genomics, In vivo, Mouse, Machine learning, CRISPR-Cas9 genome editing

## Abstract

**Background:**

Adenine base editors (ABEs) enable the conversion of A•T to G•C base pairs. Since the sequence of the target locus influences base editing efficiency, efforts have been made to develop computational models that can predict base editing outcomes based on the targeted sequence. However, these models were trained on base editing datasets generated in cell lines and their predictive power for base editing in primary cells in vivo remains uncertain.

**Results:**

In this study, we conduct base editing screens using SpRY-ABEmax and SpRY-ABE8e to target 2,195 pathogenic mutations with a total of 12,000 guide RNAs in cell lines and in the murine liver. We observe strong correlations between in vitro datasets generated by ABE-mRNA electroporation into HEK293T cells and in vivo datasets generated by adeno-associated virus (AAV)- or lipid nanoparticle (LNP)-mediated nucleoside-modified mRNA delivery (Spearman *R* = 0.83–0.92). We subsequently develop BEDICT2.0, a deep learning model that predicts adenine base editing efficiencies with high accuracy in cell lines (*R* = 0.60–0.94) and in the liver (*R* = 0.62–0.81).

**Conclusions:**

In conclusion, our work confirms that adenine base editing holds considerable potential for correcting a large fraction of pathogenic mutations. We also provide BEDICT2.0 – a robust computational model that helps identify sgRNA-ABE combinations capable of achieving high on-target editing with minimal bystander effects in both in vitro and in vivo settings.

**Supplementary Information:**

The online version contains supplementary material available at 10.1186/s13059-025-03586-7.

## Background

Adenine base editors (ABEs) enable the precise conversion of A•T to G•C nucleotides without causing DNA double-strand breaks or requiring homology-directed repair from DNA donor templates [1–3]. They are composed of laboratory-evolved *E.coli* adenosine deaminases (ecTadA) fused to nuclease-impaired Cas9 (D10A) proteins, and a single guide RNA (sgRNA) which guides the base editor complex to the desired locus in the genome [4, 5]. Among the most frequently used ABE variants are ABEmax, a fusion of Streptococcus pyogenes SpCas9(D10A) and the codon-optimized ecTadA7.10 [6], and ABE8e, in which the processivity of the adenine deaminase was further enhanced by phage assisted directed protein evolution [7]. As the targeting range of these ABE variants is constrained by the NGG protospacer-adjacent motif (PAM) requirement of SpCas9 [8–12], researchers engineered variants towards extended PAM recognition, such as SpG that recognizes NGN motifs or SpRY that recognizes NRN and to a lesser extent NYN motifs [13–21].

With these PAM-relaxed base editors at hand, nearly any site in the genome can be targeted, allowing to shift the position of the target base within the protospacer. While this strategy can be used to maximize on-target editing and minimize unintended bystander editing (conversion of neighbouring adenines) [2, 22], it requires experimental testing of different sgRNA-ABE combinations. This is a laborious and time-consuming process, making computational models that predict base editing efficiencies in silico highly valuable [23–27]. However, currently available models are only trained and tested on base editing datasets generated in vitro in cell lines, and their accuracy for predicting in vivo base editing outcomes in tissues remains uncertain [28].

To address this limitation, we conducted ABE screens not only in cell lines but also in the murine liver, and developed a machine-learning model capable of predicting editing efficiencies with high accuracy in both contexts.

## Results

### ABE screening in cell lines

**Fig. 1:**
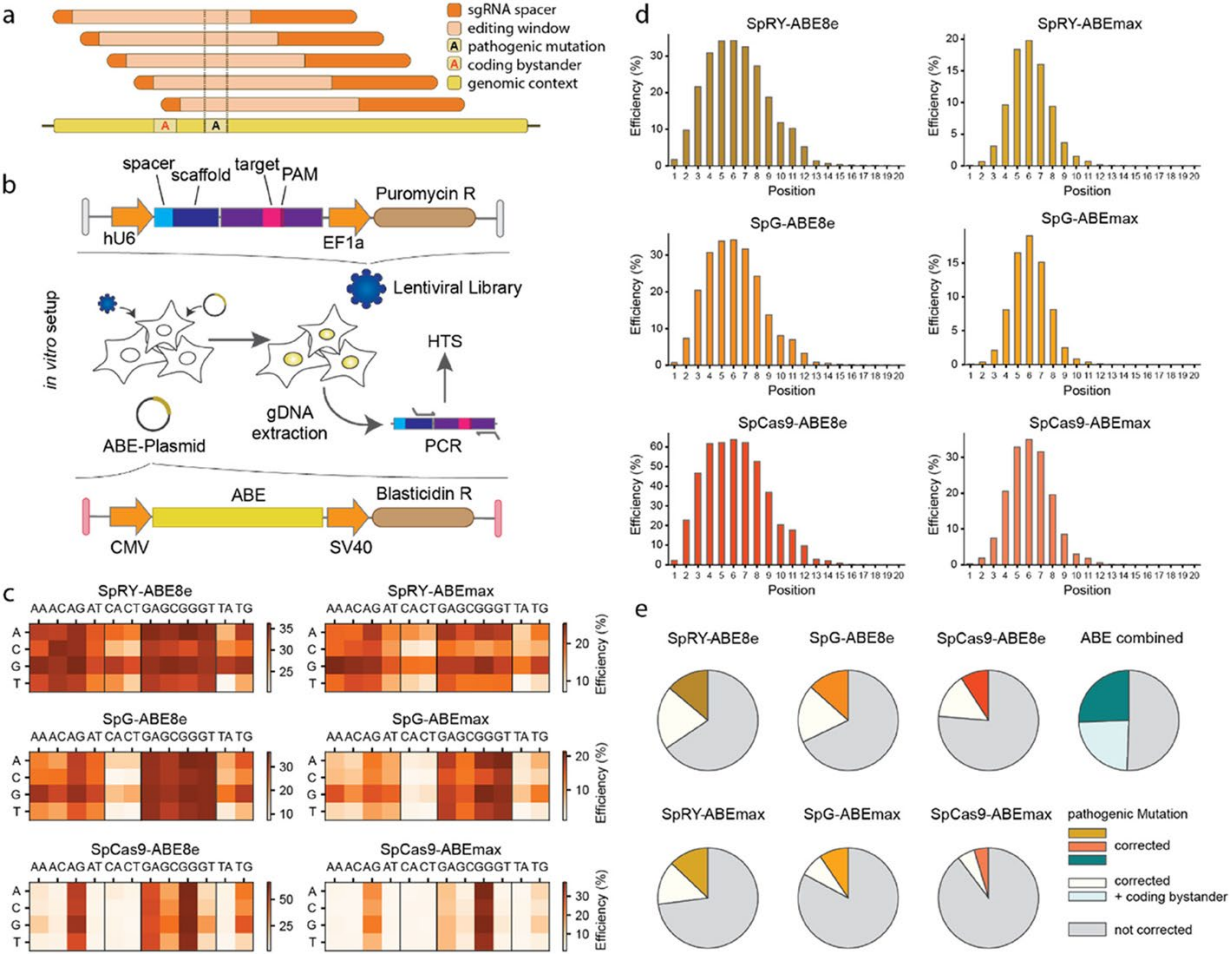
High-throughput ABE screening in HEK293T cells using target-matched sgRNA libraries. **a** Strategy of correcting pathogenic mutations without bystander editing by sgRNA tiling. The sgRNA not including the coding bystander within the editing window is shadowed darker. **b** Schematics of the ABE screen in HEK293T cells using plasmid transfection for ABE delivery. **c** Total editing efficiencies for each PAM in HEK293T cells after 10 days ABE selection with SpRY-ABE8e and SpRY-ABEmax (top row), SpG-ABE8e and SpG-ABEmax (middle row), SpCas9-ABE8e and SpCas9-ABEmax (bottom row). Y-axis indicates the 1st nucleotide of the PAM motif, the x-axis the 2nd and 3.^rd^ nucleotide of the PAM. **d** Editing window for SpRY-ABE8e and SpRY-ABEmax (top row), SpG-ABE8e and SpG-ABEmax (middle row), SpCas9-ABE8e and SpCas9-ABEmax (bottom row). Datasets were filtered for best PAMs (NRN for SpRY, NGN for SpG, and NGG for SpCas9). **e** Correction of pathogenic mutations in the library with- or without inducing non-silent bystander mutations for different base editors. Cut-offs were ≥ 10% for on-target editing and ≤ 0.5% for bystander editing. Target sites with on-target editing below 10% were defined as not corrected. Number of target sites (n) for SpRY-ABE8e: 11838, SpRY-ABEmax: 11497, SpG-ABE8e: 10287, SpG-ABEmax: 9400, SpCas9-ABE8e: 7540, SpCas9-ABEmax: 9702, ABE combined: 12000

To generate adenine base editing datasets, we performed screens in HEK293T cells, where cell pools containing target-matched sgRNA libraries were transfected with plasmids encoding for different ABE variants (Fig. 1a-b). For our library, 2,195 G-to-A or C-to-T transition point mutations causing monogenic diseases were selected from the ClinVar [29] and LOVD [30] databases. Depending on the availability of SpRY-compatible PAM sequences (NRN, NCW and NTR), each mutation was targeted by up to six sgRNAs, enabling us to shift the target base between positions 2 to 12 of the protospacer (Fig. 1a). Oligonucleotides containing sgRNA sequences paired with their target sites (20-nt protospacer flanked with the genomic context) were synthesized and cloned into a lentiviral vector for transduction into HEK293T cells at an MOI of 0.3 (Fig. 1b). Subsequently, cells were transfected with different ABE variants (SpCas9-ABEmax, SpCas9-ABE8e, SpG-ABEmax, SpG-ABE8e, SpRY-ABEmax, and SpRY-ABE8e), and cultured under selection for either 5 or 10 days prior to genomic DNA extraction and high-throughput amplicon sequencing (HTS).

We observed high library coverage and a strong correlation of editing rates between the three biological replicates after data processing and filtering (Additional file 1: Figs. S1 and S2). Additionally, we noted a strong correlation in base editing outcomes between the datasets with 5 and 10 days of selection (Spearman’s *R* = 0.78–0.97, and Pearson’s *r* = 0.9–0.96; Fig. S1b), prompting us to focus only on the 10 days dataset for further analysis (termed HEK-Plasmid dataset). When we first assessed PAM preferences of the different ABE variants, we found that they closely resembled those of the respective Cas9 nuclease variants (Fig. 1c) [13]. Specifically, SpRY-ABE variants achieved editing on all NRN and to a lesser extend NYN motifs, while SpG-ABE variants were primarily limited to NGN and NAN PAMs and SpCas9-ABE variants were restricted to NGG and NAG PAMs.

To next evaluate the average editing efficiencies of different base editor variants, we filtered the datasets based on the preferred PAM sequences of the Cas9 variants: NRN for SpRY, NGN for SpG, and NGG for SpCas9. Subsequent high-throughput sequencing (HTS) analysis revealed higher average editing rates for SpCas9 ABEs on NGG PAMs (36.0% for SpCas9-ABEmax and 64.9% for SpCas9-ABE8e) compared to SpG and SpRY ABEs on NGN or NRN PAMs, respectively (17.4% for SpG-ABEmax, 34.2% for SpG-ABE8e, 20.2% for SpRY-ABEmax and 33.9% for SpRY-ABE8e; Additional file 1: Fig. S2b, c).

Consistent with previous findings, the analysis of A-to-G conversions across the entire protospacer showed an editing window of approximately 7 bases for ABEmax and 11 bases for ABE8e variants (Fig. 1d) [1, 7, 13]. Consequently, correction of pathogenic mutations often included bystander editing. Since coding bystander mutations can be problematic, especially for translational applications, we evaluated the frequency at which pathogenic mutations could be corrected without inducing bystanders with at least one of the sgRNA-ABE combinations. Our analysis revealed that among the 36.9% of pathogenic A-to-G mutations that could be corrected with efficiencies above 10%, 69.4% did not exhibit bystander editing using a cut-off of ≤ 0.5% (Fig. 1e).

### ABE screening in the murine liver

**Fig. 2:**
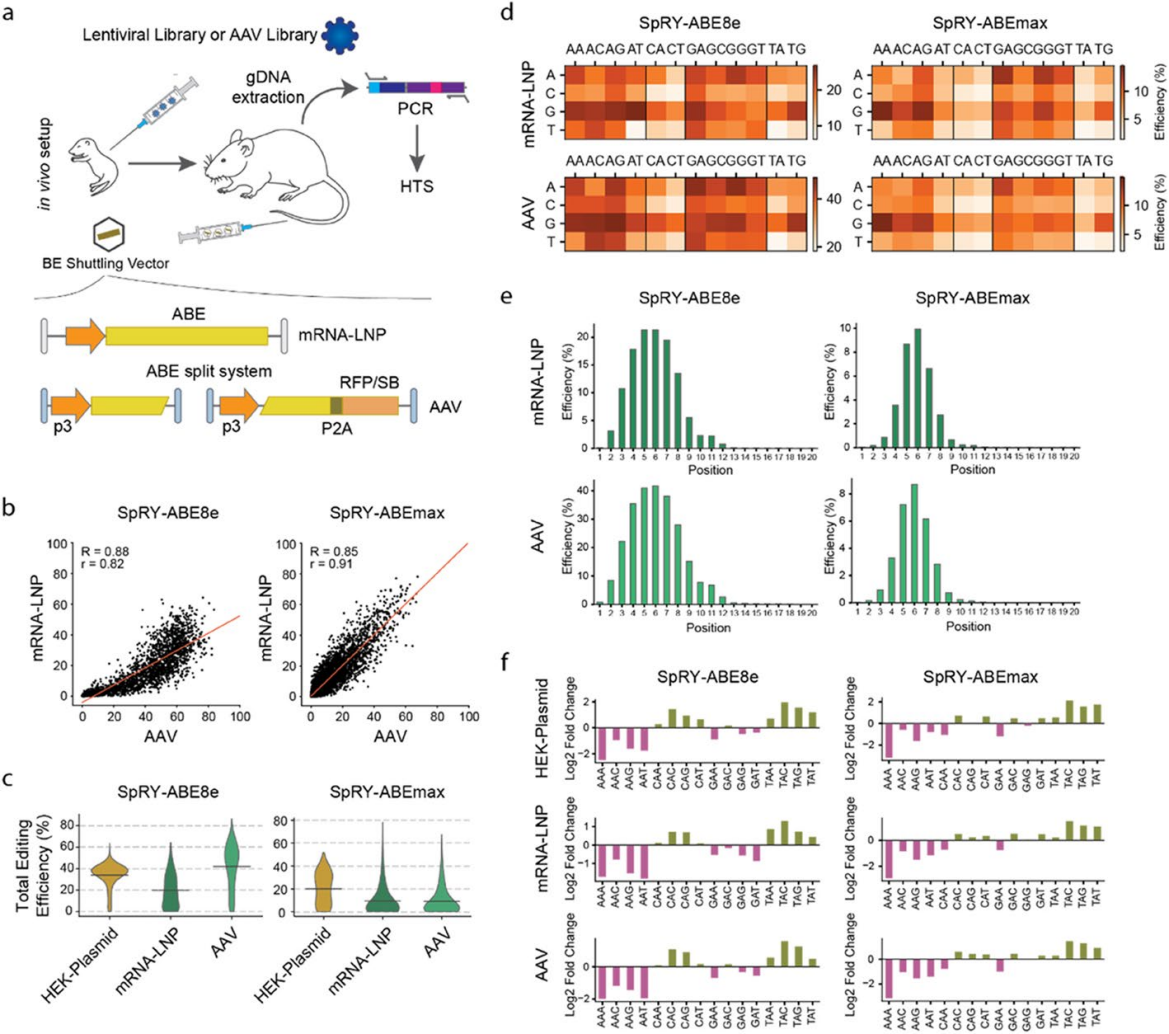
High-throughput ABE screening in the liver cells with target-matched sgRNA libraries reveals correlation to cell culture.** a** The sgRNA library was injected in p1 pups prior to ABE injection in juvenile mice. Editing rates were analysed by HTS. **b** Correlation of total A-to-G editing between the mRNA-LNP and AAV dataset with SpRY-ABE8e (*n* = 2176) and SpRY-ABEmax (*n* = 7247). The red line represents linear regression.** c** Violin plot of total editing efficiency for SpRY-ABE8e and SpRY-ABEmax in the indicated datasets. Datasets were filtered for most efficient PAMs (NRN) and mean editing efficiency is plotted (grey line). n for SpRY-ABE8e = 7882, 1623, 3459 and SpRY-ABEmax = 7644, 5170, 5852. **d** Total editing efficiency for each PAM present in the library for SpRY-ABE8e (left) and SpRY-ABEmax (right) for the mRNA-LNP and AAV datasets.** e** Editing window in the mRNA-LNP and AAV datasets are for SpRY-ABE8e (left) and SpRY-ABEmax (right) filtered for best PAMs (NRN). **f** Proportion of the different tri-nucleotide motifs for loci above mean editing efficiency (top) and below mean editing efficiency (bottom) for SpRY-ABE8e (left) and SpRY-ABEmax (right) of various screening methods

To determine whether in vitro adenine base editing outcomes in cell lines are comparable to editing outcomes in primary cells in vivo, we next conducted ABE screens in the murine liver. Lentiviral vectors encoding for the target-matched sgRNA library were intravenously injected into new-born mice for stable genomic integration into hepatocytes [31, 32]. After six weeks mice were treated with nucleoside-modified and purified mRNA-LNP encoding for SpRY-ABE8e or SpRY-ABEmax (termed mRNA-LNP dataset), or with AAV9 vectors encoding for intein-split variants of SpRY-ABE8e or SpRY-ABEmax (termed AAV dataset; Fig. 2a) [33, 34]. One week after mRNA-LNP treatment or six weeks after AAV treatment hepatocytes were isolated and editing rates were analysed by HTS. Our results revealed strong correlations between individual replicates (*R* = 0.79—0.88; Additional file 1: Fig. S3a, b) and between the AAV- and mRNA-LNP datasets (*R* = 0.88 for SpRY-ABE8e and *R* = 0.85 for SpRY-ABEmax; Fig. 2b). Moreover, in both datasets the mean editing efficiencies were higher for SpRY-ABE8e (41.9% for the AAV dataset and 19.7% for the mRNA-LNP dataset) compared to SpRY-ABEmax (9.3% for the AAV dataset and 9.5% for the mRNA-LNP dataset) (Fig. 2c), and features such as PAM recognition, the width of the editing window, or the preference for specific tri-nucleotide motifs (nucleotides flanking the target base) did not differ (Fig. 2d-f). Of note, we also conducted ABE screens in mice where the target-matched sgRNA library was delivered via AAV vectors and genomically integrated into hepatocyte genomes via Sleeping Beauty (SB) transposition (see methods section for details) [35, 36]. Supporting our results from the screens with the lentiviral target-matched sgRNA library, these experimental changes did not affect the distribution of editing outcomes (Additional file 1: Figs. S3c-d and S4).

**Fig. 3:**
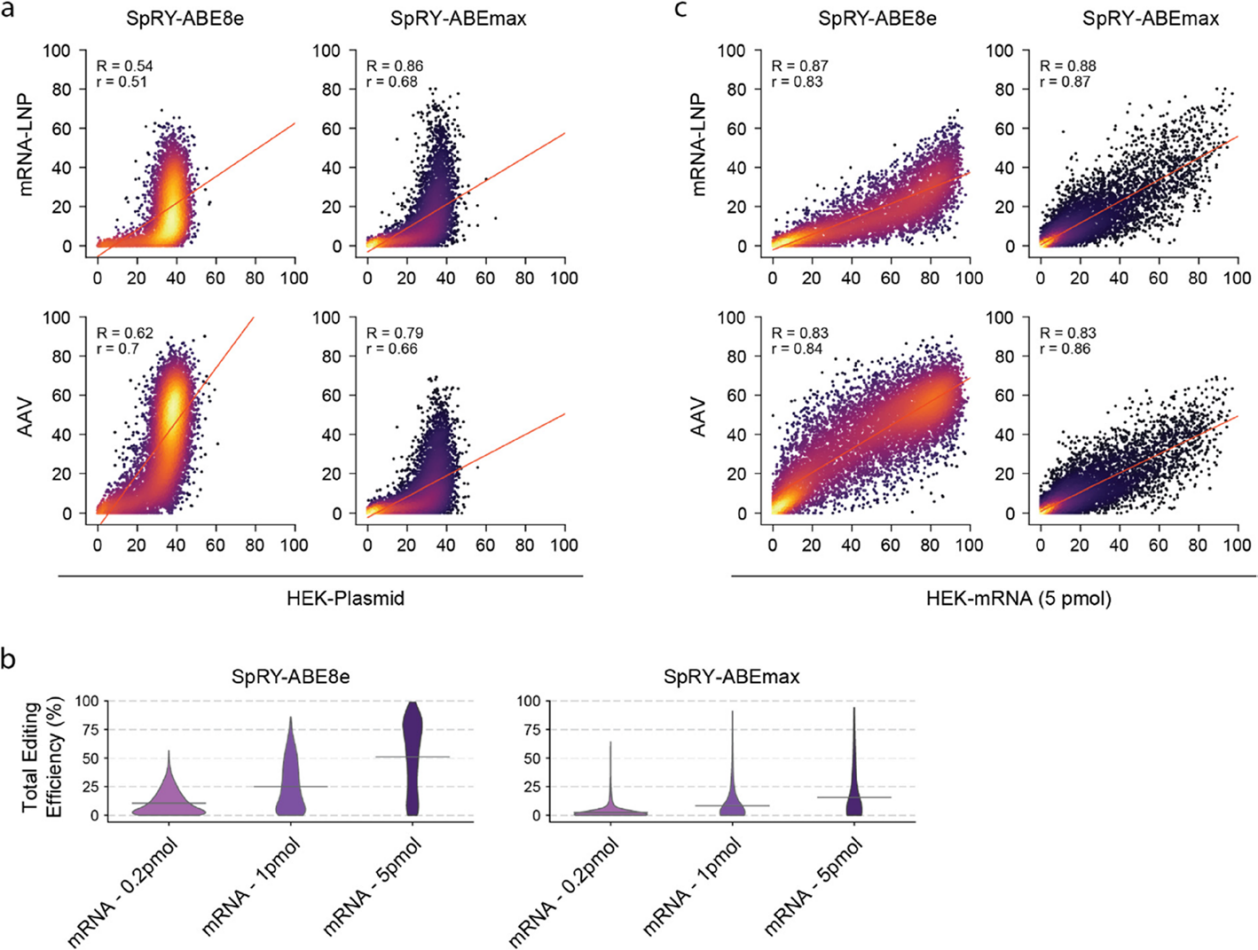
Correlation of editing efficiencies between in vitro and in vivo ABE screening datasets.** a** Correlation of total A-to-G editing efficiency between in vivo (mRNA-LNP and AAV) and in vitro (HEK-Plasmid) screening datasets for SpRY-ABE8e (left, *n* = 2418, 5233) and SpRY-ABEmax (right, *n* = 7817, 8770). **b** Violin plots of total editing efficiency in mRNA-ABE datasets with SpRY-ABE8e (top) and SpRY-ABEmax (bottom) with 0.2 pmol, 1 pmol or 5 pmol mRNA transfection. Datasets were filtered for best PAMs (NRN) and mean editing efficiency is given (grey line). n for SpRY-ABE8e = 6361, 6424, 5730 and SpRY-ABEmax = 6159, 6322, 5961.** c** Correlation of total A-to-G editing efficiency between in vivo (mRNA-LNP and AAV) and in vitro (HEK-mRNA) screening datasets for SpRY-ABE8e (left, *n* = 2388, 5018) and SpRY-ABEmax (right, *n* = 7308, 7897). The red line in all plots represents linear regression

When we next compared results from our in vivo screens to our in vitro screen (HEK-Plasmid dataset), we obtained substantially weaker correlations (*R* = 0.54–0.63 for SpRY-ABE8e and 0.79–0.86 for SpRY-ABEmax; Fig. 3a, Additional file 1: Fig. S5a). This led us to investigate whether certain sequence-derived features such as melting temperature, GC content and DeepSpCas9 score influenced editing outcomes differently in in vitro and in vivo experiments. However, neither linear correlation analysis (Additional file 2: Table S1-5), nor SHAP (Shapley Additive exPlanations [37]) analysis on XGBoost regression models trained to predict editing outcomes revealed significant differences between in vitro and in vivo datasets (Additional file 1: Fig. S5b, c). We then revisited the base editing datasets and noticed a significant difference in the distribution of editing efficiencies in vitro and in vivo*,* with an accumulation of editing efficiencies at around 40% for the SpRY-ABE8e and 30% for the SpRY-ABEmax in the HEK-Plasmid datasets (Figs. 2c and 3a). Speculating that this skew could be caused by a saturation of editing rates in the cells that express the base editor, we adapted the in vitro screening protocol and electroporated the HEK293T cell pool containing the lentiviral library with either 0.2 pmol, 1 pmol or 5 pmol of mRNA encoding for SpRY-ABE8e or SpRY-ABEmax (termed HEK-mRNA dataset). Three days post-electroporation, DNA was extracted and editing rates were analysed by HTS. We observed high Spearman correlations between replicates across the three mRNA-ABE concentrations, ranging from 0.91 to 0.98 for SpRY-ABE8e and 0.69 to 0.94 for SpRY-ABEmax (Additional file 1: Fig. S6). Moreover, while median editing rates increased with the mRNA dose, ranging from 25.0% at 0.2 pmol to 51.2% at 5 pmol for SpRY-ABE8e and from 8.3% at 0.2 pmol to 15.5% at 5 pmol for SpRY-ABEmax, they were more evenly distributed compared to the HEK-Plasmid dataset (Fig. 3b). When we then compared the in vitro HEK-mRNA dataset to the in vivo datasets, we observed a substantial increase in correlations for SpRY-ABE8e as well as SpRY-ABEmax (mRNA-LNP: *R* = 0.87 and 0.88, *r* = 0.83 and 0.87; AAV: *R* = 0.83 and 0.83, *r* = 0.84 and 0.86; Fig. 3b, Additional file 1: Fig. S7).

### Development of a deep learning model for predicting adenine base editing

Next, we utilized the ABE datasets to develop and train computational models for predicting adenine base editing efficiencies. We adapted the BE-DICT model architecture [25] by changing its design from an encoder-decoder to an encoder-encoder neural network [38], reducing its computational complexity (denoted as BEDICT1.2). The new model takes both, the reference sequence (the target sequence) and an output sequence (each potential editing outcome) as input and estimates the probability of obtaining this output sequence, substantially decreasing the computation time of the model.

To determine which parts of the target sequences are crucial for predicting editing efficiencies, we trained the model on the HEK-Plasmid datasets (80% train, 10% test, 10% validation; split performed on a gene level to avoid information leakage between nearby pathogenic sequences) using three different input configurations: either only the 20nt protospacer, the protospacer plus the 4nt PAM, or the protospacer plus the 4nt PAM and plus 5nt flanking sequences (Additional file 1: Fig. S8a). As expected, including the PAM in the input significantly improved the predictive accuracy of the model, whereas including the flanking sequences did not affect the model performance. Consequently, we restricted the input sequence to the protospacer plus the PAM.

**Fig. 4:**
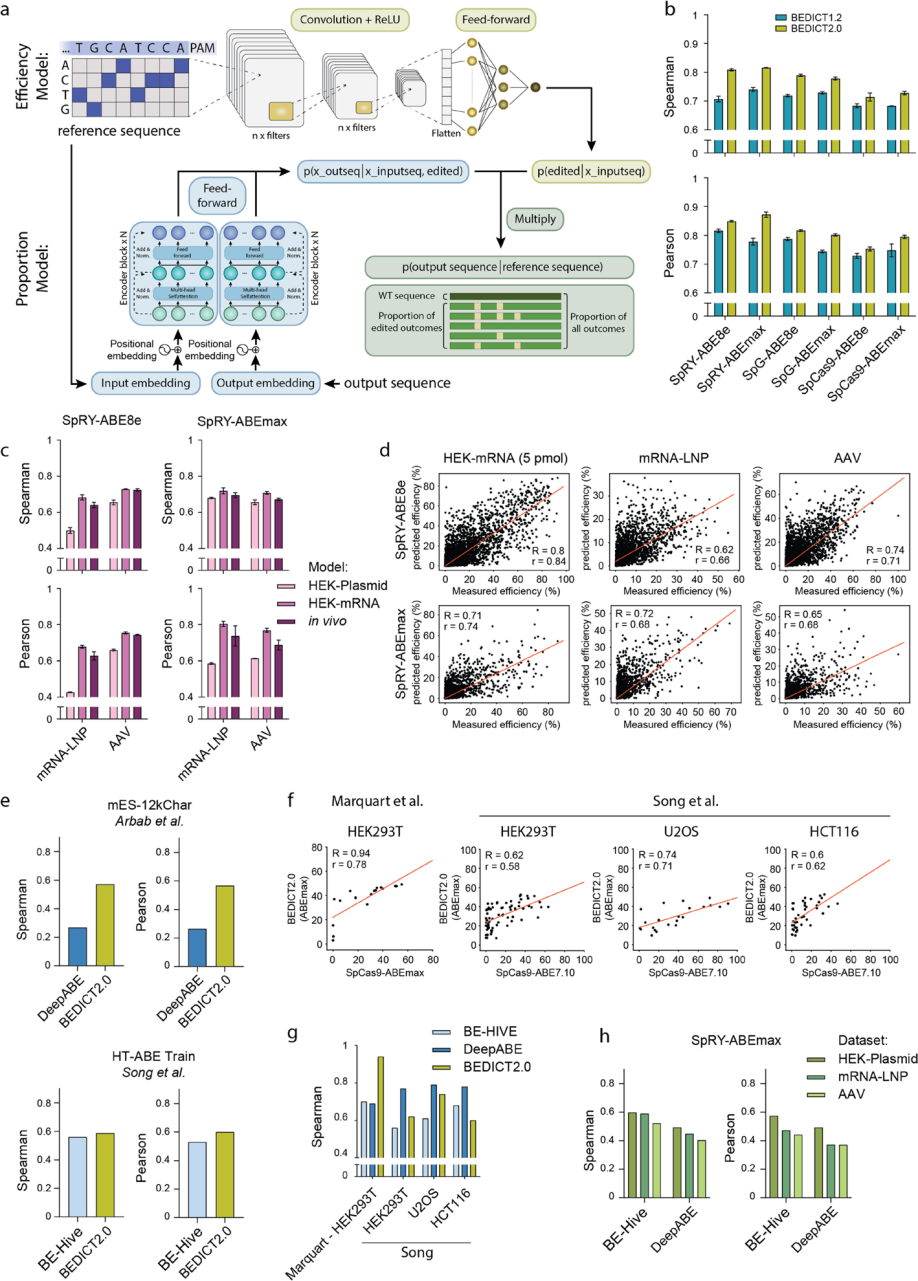
Establishment and evaluation of BEDICT2.0, a machine learning model predicting ABE activity in vitro and in vivo. **a** Schematics of BEDICT2.0 machine learning algorithm. BEDICT2.0 includes an Efficiency Model (predicts total editing efficiency) and a Proportion Model (predicts distribution within the edited reads). Outputs of both models are combined to predict editing efficiency. **b** Comparison of the performance of BEDICT1.2 or BEDICT2.0 on various HEK-Plasmid test datasets generated in this study. **c** Comparison of the performance of BEDICT2.0 trained on either HEK-Plasmid and tested on the in vivo datasets, trained on HEK-mRNA and tested on the in vivo datasets or trained and tested on the in vivo datasets. **d** Editing efficiency predicted by BEDICT2.0 plotted against the measured efficiency for SpRY-ABE8e (top) and SpRY-ABEmax (bottom) for HEK-mRNA (5 pmol), mRNA-LNP or AAV datasets. The red line represents linear regression. **e** Comparison of BEDICT2.0 to other base editing prediction models on adenine base editing datasets from target-matched sgRNA library screens. Datasets used for comparison are SpCas9-ABEmax (mES-12kChar) [23] and SpCas9-ABE7.10 (HT-ABE Train) [24]. ML-models used for predicting ABE editing outcome: DeepABE [24], BE-Hive-ABE-HEK293T [23] and BEDICT2.0 (this study). **f** Total A-to-G editing efficiency at endogenous loci in various datasets correlated to BEDICT2.0 (trained on the HEK-plasmid dataset) predictions. n for Marquart-HEK293T [25]: 18, Song-HEK293T: 72, Song-U2OS: 22, Song-HCT116: 41 [24]. **g** Spearman and Pearson correlation of measured and predicted editing efficiencies with BE-HIVE [23], DeepABE [24] and BEDICT2.0 (trained on HEK-plasmid) on various datasets generated on endogenous loci. **h** Spearman and Pearson correlation of measured and predicted editing efficiencies of BE-HIVE [23] and DeepABE [24] on the different SpRY-ABEmax datasets. Datasets were filtered for protospacers with NGG PAMs for DeepABE, as the model can only be applied for NGG PAMs

During training, we noticed that BEDICT1.2 predominantly focuses on accurately predicting the unedited sequences, as these typically constitute a high proportion of all reads at the target sites (most target sites contain multiple A bases, leading to various combinations of edited outcomes at low frequencies). To address this issue, we split the BEDICT1.2 model into two distinct components: one dedicated to predicting the total editing efficiency (combination of all edited reads—Efficiency Model) and the other dedicated to estimating the distribution within the edited reads (Proportion Model). The resulting model, referred to as BEDICT2.0 (Fig. 4a, Additional file 1: Fig. S8b), combines the values of the Efficiency and Proportion models and demonstrates improved prediction accuracy compared to BEDICT1.2 when applied to the HEK-Plasmid test dataset (BEDICT2.0: SpRY-ABE8e *R* = 0.81 *r* = 0.85, SpRY-ABEmax *R* = 0.82 *r* = 0.87; Fig. 4b). However, when we next applied BEDICT2.0 to our in vivo test datasets, the performance was substantially lower (SpRY-ABE8e: *R* = 0.5 and *r* = 0.43 for mRNA-LNP and *R* = 0.66 and *r* = 0.66 for AAV; SpRY-ABEmax: *R* = 0.68, *r* = 0.59 for mRNA-LNP and *R* = 0.66, *r* = 0.61 for AAV; Fig. 4c). Consequently, we also trained BEDICT2.0 on the HEK-mRNA dataset, which compared to the HEK-plasmid dataset showed a higher correlation to the in vivo datasets (Fig. 3a,b). This resulted in increased performance, comparable to BEDICT2.0 models that were directly trained on the in vivo datasets (SpRY-ABE8e: *R* = 0.68, *r* = 0.68 tested on mRNA-LNP, *R* = 0.73, *r* = 0.75 tested on AAV; SpRY-ABEmax: *R* = 0.72, *r* = 0.8 tested on mRNA-LNP, *R* = 0.71, *r* = 0.77 tested on AAV; Fig. 4c,d, Additional file 1: Fig. S8c,d).

To investigate whether increasing the size of the base editing dataset could further enhance the prediction accuracy of BEDICT2.0, we subdivided the HEK-mRNA datasets into bins of varying sizes, ranging from 10 to 100% of the total data (Additional file 1: Fig. S8e). Our analysis revealed that larger dataset bins improved editing prediction accuracy. However, the improvements plateaued, suggesting that further increasing the library size would result in only marginal gains in the prediction accuracy of BEDICT2.0.

We then benchmarked the performance of BEDICT2.0 to other machine learning models designed to predict base editing outcomes with ABEmax, including BE-HIVE [23], a deep conditional autoregressive model, and DeepABE [24], a convolutional neural network (CNN) model (Additional file 3: Table S1). To minimize the influence of experimental biases, we compared BEDICT2.0 to BE-HIVE using the DeepABE training dataset, and BEDICT2.0 to DeepABE using the BE-HIVE training dataset. In these comparisons, BEDICT2.0 performed slightly better than DeepABE and comparable to BE-HIVE (Fig. 4e). Subsequently, we tested all three models on datasets in which SpCas9 ABEmax was used to edit endogenous target sites (Song et al., 2020 [24] and Marquart et al., 2021 [25]). While BEDICT2.0 showed a slight advantage on the dataset generated in our laboratory (Marquart et al. [25]) and DeepABE on the dataset generated in their laboratory (Song et al. [24]), overall the performance of the three models was comparable (Fig. 4f,g, Additional file 1: Fig. S8f).

BE-HIVE and DeepABE were trained on in vitro cell line datasets in which the base editor was delivered via plasmid. Therefore, we examined whether their performance, like that of BEDICT2.0 (trained on the HEK-plasmid dataset), would also decrease in in vivo ABEmax datasets. Applying both models to the HEK-plasmid, mRNA–LNP, and AAV SpRY-ABEmax datasets revealed that their performance dropped from Spearman and Pearson of *R* = 0.6, *r* = 0.57 (BE-HIVE) and *R* = 0.49, *r* = 0.49 (DeepABE) in the HEK-plasmid dataset to *R* = 0.52–0.59, *r* = 0.44–0.47 (BE-HIVE) and *R* = 0.4–0.45, *r* = 0.37–0.43 (DeepABE) in the in vivo datasets (Fig. 4h).

## Discussion

In this study, we performed high-throughput screens to systematically evaluate the efficiency and accuracy of adenine base editors (ABEs) in correcting pathogenic mutations. We combined six ABE variants (SpCas9-ABEmax, SpG-ABEmax, SpRY-ABEmax, SpCas9-ABE8e, SpG-ABE8e, and SpRY-ABE8e) with 12,000 different sgRNAs to target more than 2,000 pathogenic mutations. In HEK293T cells, these screens revealed that approximately 25% of the targeted pathogenic mutations could be corrected with efficiencies above 10% and no detectable bystander editing for at least one ABE–sgRNA combination. Moreover, although SpRY-based ABEs enabled editing at a far broader range of PAMs than SpCas9- or SpG-based ABEs, their average on-target editing efficiencies were lower. These findings recapitulate a trade-off previously documented in Cas9 nuclease studies [39], in which a broader target scope often accompanies a reduction in average editing rates.

We next assessed how different delivery modalities and cell types influence base-editing outcomes. Screening the same sgRNA library with SpRY-ABEmax or SpRY-ABE8e delivered into the murine liver via AAV or mRNA-LNP, we observed minimal differences in the distribution of editing outcomes between these two delivery methods. In contrast, in vivo results correlated less strongly with datasets derived from plasmid-based transfection of ABEs into HEK293T cells. However, when we adapted our in vitro protocol to deliver the base editor into HEK cells via mRNA electroporation rather than plasmid transfection, the correlations with in vivo datasets improved substantially. A likely explanation is that mRNA delivery better recapitulates the physiological expression levels of the editor after in vivo delivery—with high ABE expression after plasmid transfection resulting in saturating editing rates at many target sites, obscuring meaningful differences among sgRNAs at these loci. Consistent with this hypothesis, we have previously observed that ABE expression in HEK cells after plasmid transfection can be more than 10,000-fold higher than after in vivo delivery via AAV or mRNA–LNP [28].

Building on our comprehensive base-editing datasets, we next developed and validated a deep learning model, BEDICT2.0, to predict ABE editing efficiencies. When trained on plasmid-based cell-line datasets, BEDICT2.0 performed on par with previously developed machine-learning models, such as BE-HIVE and DeepABE, on external datasets. However, as with these earlier models, its performance decreased when applied to in vivo datasets. We therefore also trained BEDICT2.0 on cell line data where ABE was delivered via mRNA, which resulted in a model that maintained high accuracy in vivo.

## Conclusion

In conclusion, our work confirms that adenine base editing holds considerable potential for correcting a large fraction of pathogenic mutations. We also provide BEDICT2.0 – a robust computational model that helps identify sgRNA-ABE combinations capable of achieving high on-target editing with minimal bystander effects in both in vitro and in vivo settings.

## Methods

### Oligo library design

2100 Target loci were selected via ClinVar [29] and LOVD [30] database (August 2020; ‘Pathogenic’ and ‘Likely Pathogenic’ mutations, monogenic disorders, G-to-A conversion mutation targetable by ABE). In order to find PAM compatible with SpRY, genomic region flanking the target site were extracted from UCSC server (http://genome.ucsc.edu/) and scanned for NGN, NAN, NCA, NCT, NTA and NTG PAM. For each target loci, 5–6 sgRNA were selected in such a way, that the target base is at position 2–12 (starting from position 6, then 5 and 7, 4 and 8, 3 and 9, 2 and 10, 11 and 12), summarizing in a total of 12′000 sgRNA. The custom oligonucleotide was purchased from Twist Bioscience, including the following elements: G/20N spacer, SpCas9 optimized scaffold [40, 41], corresponding target locus containing the 3 nt PAM and 30 nucleotides overhang on each site of the complementary region to the spacer binding site.

### Cloning of plasmids

All plasmids were either generated using isothermal assembly (NEBuilder® HiFi DNA Assembly Cloning Kit, NEB) or restriction digest and ligation using T4 ligase (NEB). PCR were conducted using NEBNext® High-Fidelity 2X PCR Master Mix (NEB).

Plasmids p2T-CMV-ABEmax -BlastR (Addgene #152989) and ABE8e (Addgene #138489) were gifts from David Liu. Plasmid p2T-ABE8e-SpCas9-BlastR was generated by ligation of the ABE8e transgene (AgeI-NotI digest of pCMV-ABE8e) into the Tol2 compatible backbone (AgeI-NotI-EcoRV digest of p2T-CMV-ABEmax-BlastR). Plasmids p2T-CMV-ABEmax-SpG-BlastR and p2T-CMV-ABE8e-SpG-BlastR were generated by isothermal assembly of either PCR amplified ABEmax or ABE8e with SpG transgene into the Tol2 compatible backbone. Plasmids p2T-CMV-ABEmax-SpRY-BlastR and p2T-CMV-ABE8e-SpRY-BlastR were generated by isothermal assembly of either PCR amplified ABEmax or ABE8e with SpRY transgene into the Tol2 compatible backbone.

Lenti-gRNA-p3-eGFP was generated by PCR amplified p3 and eGFP transgene into the Lenti compatible backbone (ApaI-MluI digest of Lenti-gRNA-puro, a gift from Hyongbum Kim [42], Addgene #84752).

AAV-library plasmid was generated as following: Linearized pcDNATM3.1/Zeo( +) plasmid (BglII-XbaI, V86020 ThermoFisher) and PCR Amplified hSyn1-eGFP-WPRE-bGHp(A)-229 transgene together with the PCR amplified U6 promoter and plasmid-library cloning site were combined by isothermal assembly. The generated plasmid was further linearized (NotI-XbaI digest) and ligated into the AAV compatible backbone (NotI-XbaI digest of an AAV plasmid (p3_NLS-(1–1153)-GG)). In a third and fourth step, PCR amplified RORI and LILO fragments from the template plasmid pT2/PGK-neo were cloned inside the newly generated plasmid by isothermal assembly, leading to the final cloned plasmid AAV-RORI-hSyn1-chl-GFP-WPRE-bGHp(A)-hU6-LILO (short: AAV-SB-Library plasmid).

All AAV-BE plasmids were generated by isothermal assembly of combinations of PCR amplified BE split (N-split: 1–573, C-split: 574–1368) transgene, PCR amplified p3 promoter, PCR amplified P2A as well as PCR amplified RFP or SB (PCR amplified from pCMV(CAT)T7-SB100, was a gift from Zsuzsanna Izsvak [43], Addgene #34879) into an AAV compatible backbone, over several steps.

### Plasmid-library preparation

For plasmid-library preparation the protocol described by Marquart et al. [25] was followed with minor changes to optimize the workflow. The oligonucleotide pool was PCR-amplified in 12 cycles (Primers stated in Supplemental Information) and Q5 High-Fidelity DNA Polymerase (New England Biolabs, NEB) following the manufacturer’s instructions. The resulting amplicons were gel purified using NucleoSpin Gel and PCR Clean-up Mini kit (Macherey–Nagel) following the manufacturer’s instructions. Lenti-gRNA-puro, Lenti-sgRNA-p3-eGFP or AAV-SB-Library were digested with Esp3I restriction enzyme and Shrimp Alkaline Phosphatase (rSAP, NEB) for 12 h at 37°C. After gel purification, the oligo-pool amplicons were assembled into the linearized Lenti-gRNA-Puro Lenti-sgRNA-p3-eGFP or AAV-SB-Library plasmid using NEBuilder HiFi DNA Assembly Master Mix (NEB) for 1 h at 50°C. The product was further purified by isopropanol precipitation using one volume of isopropanol, 0.02 volume 5 M NaCl and 0.01 volume GlycoBlue coprecipitant (Invitrogen). After precipitation and ethanol wash, the air-dried pellet was resuspended in dH2O. 100 ng of plasmid library were transformed per 25 µL electrocompetent cells (ElectroMAX Stbl4, Invitrogen) using a GenePulser II device (Bio-Rad). Transformed cells were recovered in S.O.C. media and incubated for 14 h at 30°C. Colonies were scraped, pooled and let grow in bacterial media for another 6 h before plasmids were purified using a Plasmid Maxiprep kit (Qiagen).

### Cell culture

HEK293T (ATCC CRL-3216) were maintained in DMEM plus GlutaMax (Thermo Fisher Scientific), supplemented with 10% (vol/vol) fetal bovine serum (FBS, Sigma-Aldrich) and 1 × penicillin–streptomycin (Thermo Fisher Scientific) at 37°C and 5% CO2. Cells were maintained at confluency below 90% and passaged every 2–3 days. N2A () were maintained in EMEM plus GlutaMax (Thermo Fisher Scientific), supplemented with 10% (vol/vol) fetal bovine serum (FBS, Sigma-Aldrich) and 1 × penicillin–streptomycin (Thermo Fisher Scientific) at 37°C and 5% CO2. Cells were maintained at confluency below 90%, passaged every 2–3 days and tested negative for *Mycoplasma* contamination. Cells were authenticated by the supplier by short tandem repeat analysis.

### Packaging of guide RNA library into lentivirus

HEK293T cells were used for lentivirus production. 2.65 µg pCMV-VSV-G (a gift from B. Weinberg [44], Addgene #8454), 5.3 µg psPAX2 (a gift from D.Trono, Addgene #12260) and 10.8 µg target library plasmid were mixed in 506 µL Opti-MEM (Thermo Fisher Scientific). After addition of 152 µL polyethyleneimine (PEI, 1 mg/mL), the transfection mix was vortexed for 10 s and incubated 10 min, before added gently to the cells at 70–80% confluency together with 25 mL serum-free DMEM. After 1 day the medium was changed to culture medium and 2 days later, supernatant was harvested. Prior to ultracentrifugation (20′000xg, 2 h), medium was filtered using a Filtropur S 0.4 (Sarstedt) filter. Lentivirus aliquots were stored at -80 °C until use.

### Pooled base editor screens

Lentivirus containing sgRNA-pool were transduced at a MOI of 0.2 and a calculated coverage of 1000 cells per gRNA in HEK293T cells at a confluence of 70–80%. One day after transduction, cells were split and selected with 2.5 µg/mL puromycin for 10 days. Selected HEK293T cells were frozen and for each new screen thawed with a coverage of 2000x. Respective base editor plasmid (9.25 ug) and helper plasmid (9.25 ug of pCMV-Tol2, a gift from Stephen Ekker [45], Addgene #31,823) were transfected in a 1:3 DNA:PEI ratio per T175 flask at a coverage of 2000x. One day after transfection, cells were split and selected with 2.5 µg/mL puromycin and 7.5 µg/mL blasticidin for 5 or 10 days. Cells were detached and genomic DNA was extracted using a Blood & Cell Culture DNA Maxi kit (Qiagen) according to the manufacturer’s instructions.

Nucleofections of HEK293T cells were performed using the NeonTM transfection system using 100 µL tips. Cells were harvested and washed 3 × with phosphate-buffered saline (PBS) prior to counting. Cells were repeatedly spun down and resuspended in R buffer (DPBS supplemented with 1 mM MgCl2 and 250 mL Sucrose) to a concentration of ~ 3 × 10^4^ cells/µL. Reactions were prepared in PBS by the respective addition of mRNA for 0.2 pmol, 1 pmol and 5 pmol. For mRNA, one pulse of 1400 mV and 20 mS pulse width was used. After nucleofection, Cells were maintained at confluency below 90% and passaged every 2–3 days. N2A () were maintained in EMEM plus GlutaMax (Thermo Fisher Scientific), supplemented with 10% (vol/vol) fetal bovine serum (FBS, Sigma-Aldrich) and 1 × penicillin–streptomycin (Thermo Fisher Scientific) at 37°C and 5% CO2 for 72 h prior to harvesting. Cells were detached and genomic DNA was extracted using a Blood & Cell Culture DNA Maxi kit (Qiagen) according to the manufacturer’s instructions. Modified nucleoside-containing mRNA was generated using N1mΨ-5′-triphosphate (TriLink) instead of UTP. Co-transcriptional addition of the trinucleotide cap1 analog, CleanCap (TriLink), was used to cap the in vitro transcribed mRNAs.

### AAV production

AAV vectors were either produced by the Viral Vector Facility of the Neuroscience Center Zurich or in-house. Briefly, AAV vectors were ultracentrifuged and diafiltered.

To generate Pseudotyped AAV9 vectors (AAV2/9), packaging, capsid, and helper plasmids (Addgene #112865 and #112867) were co-transfected in HEK293T cells and incubated for six days until harvest. The vectors were then precipitated using PEG and NaCl and subjected to gradient centrifugation with OptiPrep (Sigma-Aldrich) for further purification, following the previously described method. Subsequently, the concentrated vectors were obtained using Vivaspin® 20 centrifugal concentrators (VWR). Physical titres (vector genomes per milliliter, vg/mL) were determined using a Qubit 3.0 fluorometer (Thermo Fisher Scientific). AAV2/9 viruses were stored at -80°C until they were used. If required, they were diluted using phosphate-buffered saline (PBS) from Thermo Fisher Scientific.

### mRNA production and LNP encapsulation

mRNA production and LNP encapsulation were performed as previously described [46]. Briefly, coding sequences of base editors were cloned into an mRNA production plasmid, using HiFi DNA Assembly Master Mix (NEB). mRNAs were transcribed to contain 101 nucleotide-long poly(A) tails. m1Ψ-5′-triphosphate (TriLink) instead of UTP was used to generate modified nucleoside-containing mRNA. Capping of the in vitro transcribed mRNAs was performed co-transcriptionally using the trinucleotide cap1 analog, CleanCap (TriLink). mRNA was purified by cellulose (Sigma-Aldrich) purification as described [47]. All mRNAs were analysed by agarose gel electrophoresis and were stored frozen at − 20°C. The purified mRNAs were encapsulated in LNP, previously described in [28], and stored at -80°C until they were injected into mice.

### Animal studies

Animal experiments were performed in accordance with protocols approved by the Kantonales Veterinäramt Zürich (license number ZH159-20) and in compliance with all relevant ethical regulations. C57BL/6 J mice were housed in a pathogen-free animal facility at the Institute of Pharmacology and Toxicology of the University of Zurich. Mice were kept in a temperature- and humidity-controlled room on a 12-h light/dark cycle. Mice were fed a standard laboratory chow (Kliba Nafag no. 3437 with 18.5% crude protein).

Unless otherwise noted, new-born animals (P1) received 1.2 × 10^11^ (AAV; 30 µL in total) AAV vector genomes per animal and construct or full dose (Lentivirus; 30 µL in total) of lentivirus via the temporal vein. Adult mice were injected with 3 mg/kg of total RNA (LNP) or 1 × 10^12^ AAV vector genomes per animal via tail vein at 5–6 weeks of age, with total injection volumes of 120 µL. The average weights of neonatal (1 day) and adult mice (5 weeks) were 1.5 and 20 g, respectively. In case of delivering the library via lentivirus, mice were euthanized 6–8 weeks after injection or further injected with LNP or AAV. Adult mice were euthanized 1 week (LNP) or 6 weeks (AAV) after injection, if not stated otherwise. In case of delivering the library via AAV and SB, mice were euthanized 6 or 12 weeks after injection.

### Primary hepatocyte isolation

Primary hepatocytes were isolated as previously described11. In short, mice were euthanized and immediately perfused with Hank’s Buffer (Hank’s balanced salt solution (Thermo Fisher Scientific, 0.5 mM EDTA) via inferior vena cava. Mice were further perfused with digestion medium (low-glucose DMEM plus 1 × penicillin–streptomycin (Thermo Fisher Scientific), 15 mM HEPES and freshly added Liberase (Roche)) before isolated livers were gently dissociated in isolation medium (low-glucose DMEM supplemented with 10% (vol/vol) FBS plus 1 × penicillin–streptomycin (Thermo Fisher Scientific) and GlutaMax (Thermo Fisher Scientific)). Isolated Hepatocytes were filtered (100 µm filter), washed with isolation media/ PBS and further pelleted for DNA isolation.

### Library preparation for targeted amplicon sequencing of DNA

Next-generation sequencing (NGS) preparation of genomic DNA was performed as previously described [34]. Briefly, the library was amplified from genomic DNA by a first PCR using primers containing Illumina forward and reverse adaptor sequences (See Supplementary Note for oligonucleotides used in this study). PCR was optimized for high genomic DNA input using NEBNext® UltraTM II Q5 polymerase (NEB) and a coverage of 200-1000x, depending on screening method and replicate. PCR for each replicate were pooled and gel purified, before barcodes with primer containing unique sets of pe/p7 Illumina barcodes were added in a second PCR, using Q5 High-Fidelity DNA Polymerase (NEB). PCR were pooled and cleaned through gel purification before quantification on the Qubit 4 (Invitrogen). Pooled sgRNA screens were sequenced on a NovaSeq 6000 (Illumina, 300 cycles, paired-end). Amplicon sequences were analysed using custom Python scripts.

## Supplementary Information

Additional file 1. Contains supplementary figures (Fig. S1 – Fig. S8) and additional information to BEDICT2.0 and SHAP analysis.

Additional file 2. Contains analysis of linear correlation (Table S1-S5).

Additional file 3. Contains Spearman and Pearson correlations for prediction accuracy of ML prediction models on various datasets (Table S1).

Additional file 4. Contains additional informations to primers, oligonucleotide pool, ABE sequences and editing efficiency per position for all relevant datasets of this study.

## Data Availability

Additional information on the BEDICT2.0 algorithm and SHAP analysis used in this study can be found in Additional file 1, which includes references for the machine learning [48–50]. DNA-sequencing data are available via the NCBI Sequence Read Archive (PRJNA1107439) [51]. Source data are provided with this paper (Additional file 4). We have made the source code for BEDICT2.0 used to train and evaluate the models available on GitHub [52] and Zenodo [53]. The source code for BEDICT2.0 is available under the MIT License, an OSI-approved open source license. The full license text is provided in the GitHub repository and the Zenodo archive. The web application for BEDICT2.0 is available at go.bedict.app.

## References

[1] Gaudelli NM, Komor AC, Rees HA, Packer MS, Badran AH, Bryson DI, et al. Programmable base editing of A•T to G•C in genomic DNA without DNA cleavage. Nature. 2017;551:464–71. 29160308 10.1038/nature24644 PMC5726555

[2] Rees HA, Liu DR. Base editing: precision chemistry on the genome and transcriptome of living cells. Nat Rev Genet. 2018;19:770–88. 30323312 10.1038/s41576-018-0059-1 PMC6535181

[3] Komor AC, Kim YB, Packer MS, Zuris JA, Liu DR. Programmable editing of a target base in genomic DNA without double-stranded DNA cleavage. Nature. 2016;533:420–4. 27096365 10.1038/nature17946 PMC4873371

[4] Anzalone AV, Koblan LW, Liu DR. Genome editing with CRISPR–Cas nucleases, base editors, transposases and prime editors. Nat Biotechnol. 2020;38:824–44. 32572269 10.1038/s41587-020-0561-9

[5] Anders C, Niewoehner O, Duerst A, Jinek M. Structural basis of PAM-dependent target DNA recognition by the Cas9 endonuclease. Nature. 2014;513:569–73. 25079318 10.1038/nature13579 PMC4176945

[6] Koblan LW, Doman JL, Wilson C, Levy JM, Tay T, Newby GA, et al. Improving cytidine and adenine base editors by expression optimization and ancestral reconstruction. Nat Biotechnol. 2018;36:843–6. 29813047 10.1038/nbt.4172 PMC6126947

[7] Richter MF, Zhao KT, Eton E, Lapinaite A, Newby GA, Thuronyi BW, et al. Phage-assisted evolution of an adenine base editor with improved Cas domain compatibility and activity. Nat Biotechnol. 2020;38:883–91. 32433547 10.1038/s41587-020-0453-z PMC7357821

[8] Deveau H, Barrangou R, Garneau JE, Labonté J, Fremaux C, Boyaval P, et al. Phage response to CRISPR-encoded resistance in Streptococcus thermophilus. J Bacteriol. 2008;190:1390–400. 18065545 10.1128/JB.01412-07 PMC2238228

[9] Mojica FJM, Díez-Villaseñor C, García-Martínez J, Almendros C. Short motif sequences determine the targets of the prokaryotic CRISPR defence system. Microbiol Read Engl. 2009;155:733–40. 10.1099/mic.0.023960-0 19246744

[10] Jinek M, Chylinski K, Fonfara I, Hauer M, Doudna JA, Charpentier E. A programmable dual-RNA–guided DNA endonuclease in adaptive bacterial immunity. Science. 2012;337:816–21. 22745249 10.1126/science.1225829 PMC6286148

[11] Jiang W, Bikard D, Cox D, Zhang F, Marraffini LA. CRISPR-assisted editing of bacterial genomes. Nat Biotechnol. 2013;31:233–9. 23360965 10.1038/nbt.2508 PMC3748948

[12] Sternberg SH, Redding S, Jinek M, Greene EC, Doudna JA. DNA interrogation by the CRISPR RNA-guided endonuclease Cas9. Nature. 2014;507:62–7. 24476820 10.1038/nature13011 PMC4106473

[13] Walton RT, Christie KA, Whittaker MN, Kleinstiver BP. Unconstrained genome targeting with near-PAMless engineered CRISPR-Cas9 variants. Science. 2020;368:290–6. 32217751 10.1126/science.aba8853 PMC7297043

[14] Kleinstiver BP, Prew MS, Tsai SQ, Topkar VV, Nguyen NT, Zheng Z, et al. Engineered CRISPR-Cas9 nucleases with altered PAM specificities. Nature. 2015;523:481–5. 26098369 10.1038/nature14592 PMC4540238

[15] Anders C, Bargsten K, Jinek M. Structural plasticity of PAM recognition by engineered variants of the RNA-guided endonuclease Cas9. Mol Cell. 2016;61:895–902. 26990992 10.1016/j.molcel.2016.02.020 PMC5065715

[16] Hu JH, Miller SM, Geurts MH, Tang W, Chen L, Sun N, et al. Evolved Cas9 variants with broad PAM compatibility and high DNA specificity. Nature. 2018;556:57–63. 29512652 10.1038/nature26155 PMC5951633

[17] Miller SM, Wang T, Randolph PB, Arbab M, Shen MW, Huang TP, et al. Continuous evolution of SpCas9 variants compatible with non-G PAMs. Nat Biotechnol. 2020;38:471–81. 32042170 10.1038/s41587-020-0412-8 PMC7145744

[18] Chatterjee P, Jakimo N, Lee J, Amrani N, Rodríguez T, Koseki SRT, et al. An engineered ScCas9 with broad PAM range and high specificity and activity. Nat Biotechnol. 2020;38:1154–8. 32393822 10.1038/s41587-020-0517-0

[19] Nishimasu H, Shi X, Ishiguro S, Gao L, Hirano S, Okazaki S, et al. Engineered CRISPR-Cas9 nuclease with expanded targeting space. Science. 2018;361:1259–62. 30166441 10.1126/science.aas9129 PMC6368452

[20] Kleinstiver BP, Pattanayak V, Prew MS, Tsai SQ, Nguyen NT, Zheng Z, et al. High-fidelity CRISPR–Cas9 nucleases with no detectable genome-wide off-target effects. Nature. 2016;529:490–5. 26735016 10.1038/nature16526 PMC4851738

[21] Tycko J, Myer VE, Hsu PD. Methods for optimizing CRISPR-Cas9 genome editing specificity. Mol Cell. 2016;63:355–70. 27494557 10.1016/j.molcel.2016.07.004 PMC4976696

[22] Lavrov AV, Varenikov GG, Skoblov MY. Genome scale analysis of pathogenic variants targetable for single base editing. BMC Med Genomics. 2020;13:80. 32948190 10.1186/s12920-020-00735-8 PMC7499999

[23] Arbab M, Shen MW, Mok B, Wilson C, Matuszek Ż, Cassa CA, et al. Determinants of base editing outcomes from target library analysis and machine learning. Cell. 2020;182:463-480.e30. 32533916 10.1016/j.cell.2020.05.037 PMC7384975

[24] Song M, Kim HK, Lee S, Kim Y, Seo S-Y, Park J, et al. Sequence-specific prediction of the efficiencies of adenine and cytosine base editors. Nat Biotechnol. 2020;38:1037–43. 32632303 10.1038/s41587-020-0573-5

[25] Marquart KF, Allam A, Janjuha S, Sintsova A, Villiger L, Frey N, et al. Predicting base editing outcomes with an attention-based deep learning algorithm trained on high-throughput target library screens. Nat Commun. 2021;12:5114. 34433819 10.1038/s41467-021-25375-z PMC8387386

[26] Pallaseni A, Peets EM, Koeppel J, Weller J, Vanderstichele T, Ho UL, et al. Predicting base editing outcomes using position-specific sequence determinants. Nucleic Acids Res. 2022;50:3551–64. 35286377 10.1093/nar/gkac161 PMC8989541

[27] Kim N, Choi S, Kim S, Song M, Seo JH, Min S, et al. Deep learning models to predict the editing efficiencies and outcomes of diverse base editors. Nat Biotechnol. 2023;42:1–14. 10.1038/s41587-023-01792-x 37188916

[28] Rothgangl T, Dennis MK, Lin PJC, Oka R, Witzigmann D, Villiger L, et al. In vivo adenine base editing of PCSK9 in macaques reduces LDL cholesterol levels. Nat Biotechnol. 2021;39:949–57. 34012094 10.1038/s41587-021-00933-4 PMC8352781

[29] Landrum MJ, Lee JM, Benson M, Brown G, Chao C, Chitipiralla S, et al. ClinVar: public archive of interpretations of clinically relevant variants. Nucleic Acids Res. 2016;44:D862–8. 26582918 10.1093/nar/gkv1222 PMC4702865

[30] Fokkema IFAC, Taschner PEM, Schaafsma GCP, Celli J, Laros JFJ, den Dunnen JT. LOVD v.2.0: the next generation in gene variant databases. Hum Mutat. 2011;32:557–63. 21520333 10.1002/humu.21438

[31] Nguyen TH, Bellodi-Privato M, Aubert D, Pichard V, Myara A, Trono D, et al. Therapeutic lentivirus-mediated neonatal in vivo gene therapy in hyperbilirubinemic gunn rats. Mol Ther. 2005;12:852–9. 16140582 10.1016/j.ymthe.2005.06.482

[32] Keys HR, Knouse KA. Genome-scale CRISPR screening in a single mouse liver. Cell Genomics. 2022;2:100217. 36643909 10.1016/j.xgen.2022.100217 PMC9835819

[33] Truong DJJ, Kühner K, Kühn R, Werfel S, Engelhardt S, Wurst W, et al. Development of an intein-mediated split–Cas9 system for gene therapy. Nucleic Acids Res. 2015;43:6450–8. 26082496 10.1093/nar/gkv601 PMC4513872

[34] Villiger L, Grisch-Chan HM, Lindsay H, Ringnalda F, Pogliano CB, Allegri G, et al. Treatment of a metabolic liver disease by in vivo genome base editing in adult mice. Nat Med. 2018;24:1519–25. 30297904 10.1038/s41591-018-0209-1

[35] Scheuermann B, Diem T, Ivics Z, Andrade-Navarro MA. Evolution-guided evaluation of the inverted terminal repeats of the synthetic transposon Sleeping Beauty. Sci Rep. 2019;9:1171. 30718656 10.1038/s41598-018-38061-w PMC6362248

[36] Ye L, Park JJ, Dong MB, Yang Q, Chow RD, Peng L, et al. In vivo CRISPR screening in CD8 T cells with AAV–Sleeping Beauty hybrid vectors identifies membrane targets for improving immunotherapy for glioblastoma. Nat Biotechnol. 2019;37:1302–13. 31548728 10.1038/s41587-019-0246-4 PMC6834896

[37] Lundberg SM, Lee S-I. A Unified Approach to Interpreting Model Predictions. Adv Neural Inf Process Syst. Curran Associates, Inc.; 2017 [cited 2024 Jul 5]. Available from: https://papers.nips.cc/paper_files/paper/2017/hash/8a20a8621978632d76c43dfd28b67767-Abstract.html.

[38] Mollaysa A, Allam A, Krauthammer M. Attention-based Multi-task Learning for Base Editor Outcome Prediction. arXiv; 2023. Available from: http://arxiv.org/abs/2310.02919. Cited 2024 Jul 11.

[39] Schmid-Burgk JL, Gao L, Li D, Gardner Z, Strecker J, Lash B, et al. Highly parallel profiling of Cas9 variant specificity. Mol Cell. 2020;78:794-800.e8. 32187529 10.1016/j.molcel.2020.02.023 PMC7370240

[40] Ma H, Wu Y, Dang Y, Choi J-G, Zhang J, Wu H. Pol III Promoters to express Small RNAs: delineation of transcription initiation. Mol Ther - Nucleic Acids. 2014;3:e161. 24803291 10.1038/mtna.2014.12 PMC4040628

[41] Chen B, Gilbert LA, Cimini BA, Schnitzbauer J, Zhang W, Li G-W, et al. Dynamic imaging of genomic loci in living human cells by an optimized CRISPR/Cas system. Cell. 2013;155:1479–91. 24360272 10.1016/j.cell.2013.12.001 PMC3918502

[42] Kim HK, Song M, Lee J, Menon AV, Jung S, Kang Y-M, et al. In vivo high-throughput profiling of CRISPR-Cpf1 activity. Nat Methods. 2017;14:153–9. 27992409 10.1038/nmeth.4104

[43] Mátés L, Chuah MKL, Belay E, Jerchow B, Manoj N, Acosta-Sanchez A, et al. Molecular evolution of a novel hyperactive Sleeping Beauty transposase enables robust stable gene transfer in vertebrates. Nat Genet. 2009;41:753–61. 19412179 10.1038/ng.343

[44] Stewart SA, Dykxhoorn DM, Palliser D, Mizuno H, Yu EY, An DS, et al. Lentivirus-delivered stable gene silencing by RNAi in primary cells. RNA N Y N. 2003;9:493–501. 10.1261/rna.2192803 PMC1370415 12649500

[45] Balciunas D, Wangensteen KJ, Wilber A, Bell J, Geurts A, Sivasubbu S, et al. Harnessing a high cargo-capacity transposon for genetic applications in vertebrates. PLoS Genet. 2006;2:e169. 17096595 10.1371/journal.pgen.0020169 PMC1635535

[46] Vadovics M, Muramatsu H, Sárközy A, Pardi N. Production and Evaluation of Nucleoside-Modified mRNA Vaccines for Infectious Diseases. In: Kramps T, editor. RNA Vaccines Methods Protoc. New York, NY: Springer US; 2024. p. 167–81. 10.1007/978-1-0716-3770-8_7. Cited 2024 Jul 30. 10.1007/978-1-0716-3770-8_7 38814394

[47] Baiersdörfer M, Boros G, Muramatsu H, Mahiny A, Vlatkovic I, Sahin U, et al. A facile method for the removal of dsRNA Contaminant from In Vitro-Transcribed mRNA. Mol Ther Nucleic Acids. 2019;15:26–35. 30933724 10.1016/j.omtn.2019.02.018 PMC6444222

[48] Vaswani A, Shazeer N, Parmar N, Uszkoreit J, Jones L, Gomez AN, Kaiser U, Polosukhin I, et al. Attention is all you need. Adv Neural Inf Process Syst. 2017;30:5998–6008.

[49] He K, Zhang X, Ren S, Sun J. Deep Residual Learning for Image Recognition. 2016 IEEE Conf Comput Vis Pattern Recognit CVPR. Las Vegas, NV, USA: IEEE; 2016. p. 770–8. Available from: http://ieeexplore.ieee.org/document/7780459/. Cited 2025 Apr 8.

[50] Ba JL, Kiros JR, Hinton GE. Layer Normalization. arXiv; 2016. Available from: http://arxiv.org/abs/1607.06450. Cited 2025 Apr 8.

[51] Kissling L, Mollaysa A, Janjuha S, Mathis N, Marquart KF, Weber Y, et al. datasets to In vitro and in vivo base editing screening. NCBI SRA; 2025. Available from: https://www.ncbi.nlm.nih.gov/sra/?term=PRJNA1107439.

[52] Kissling L, Mollaysa A, Janjuha S, Mathis N, Marquart KF, Weber Y, et al. Predicting base editing outcomes with an attention-based deep learning algorithm-GitHub. GitHub; 2025. Available from: https://github.com/uzh-dqbm-cmi/BEDICT-V2. 10.1038/s41467-021-25375-z PMC8387386 34433819

[53] Kissling L, Mollaysa A, Janjuha S, Mathis N, Marquart KF, Weber Y, et al. Predicting base editing outcomes with an attention-based deep learning algorithm - Zenodo. Zenodo; 2025. Available from: https://zenodo.org/records/15185623. 10.1038/s41467-021-25375-z PMC8387386 34433819

