# Supplementary material for: Predicting adenine base editing efficiencies in different cellular contexts by deep learning
Source: Genome Biol. 2025 May 8;26:115. doi: 10.1186/s13059-025-03586-7 (PMC12060317; doi:10.1186/s13059-025-03586-7)
Supplement: Supplementary file 1 — Additional file 1. Contains supplementary figures (Fig. S1 – Fig. S8) and additional information to BEDICT2.0 and SHAP analysis. [file 13059_2025_3586_MOESM1_ESM.pdf]

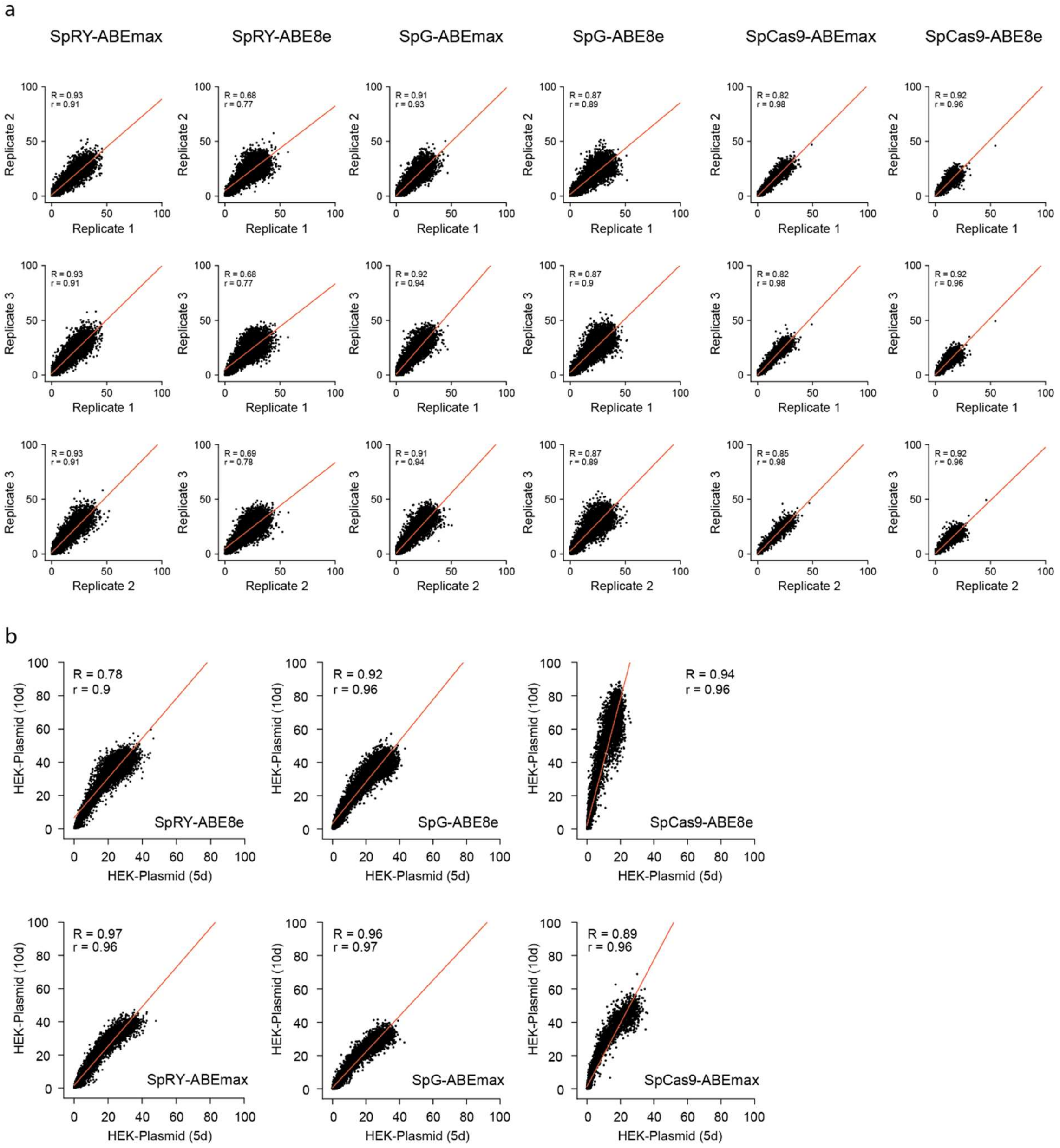

Fig. S1: Correlation of replicates of the HEK-Plasmid dataset with 5 or 10 days of ABE selection. a Scatter plots of editing efficiencies. ABEs were selected for 5 days in HEK293T cells (n for replicates of SpRY-ABEmax: 5850, SpRY-ABE8e: 7810, SpG-ABEmax: 9558, SpG-ABE8e: 9551, SpCas9-ABEmax: 11839, SpCas9-ABE8e: 11846). **b** Scatter plots of editing efficiencies. ABEs were expressed for 5 days (x-axis) or 10 days (y-axis) in HEK293T cells (n for SpRY-ABE8e: 7812, SpRY-ABEmax: 5850, SpG-ABE8e: 9419, SpG-ABEmax: 9048, SpCas9-ABE8e: 7541, SpCas9-ABEmax: 9702). The red line in all plots represents linear regression.

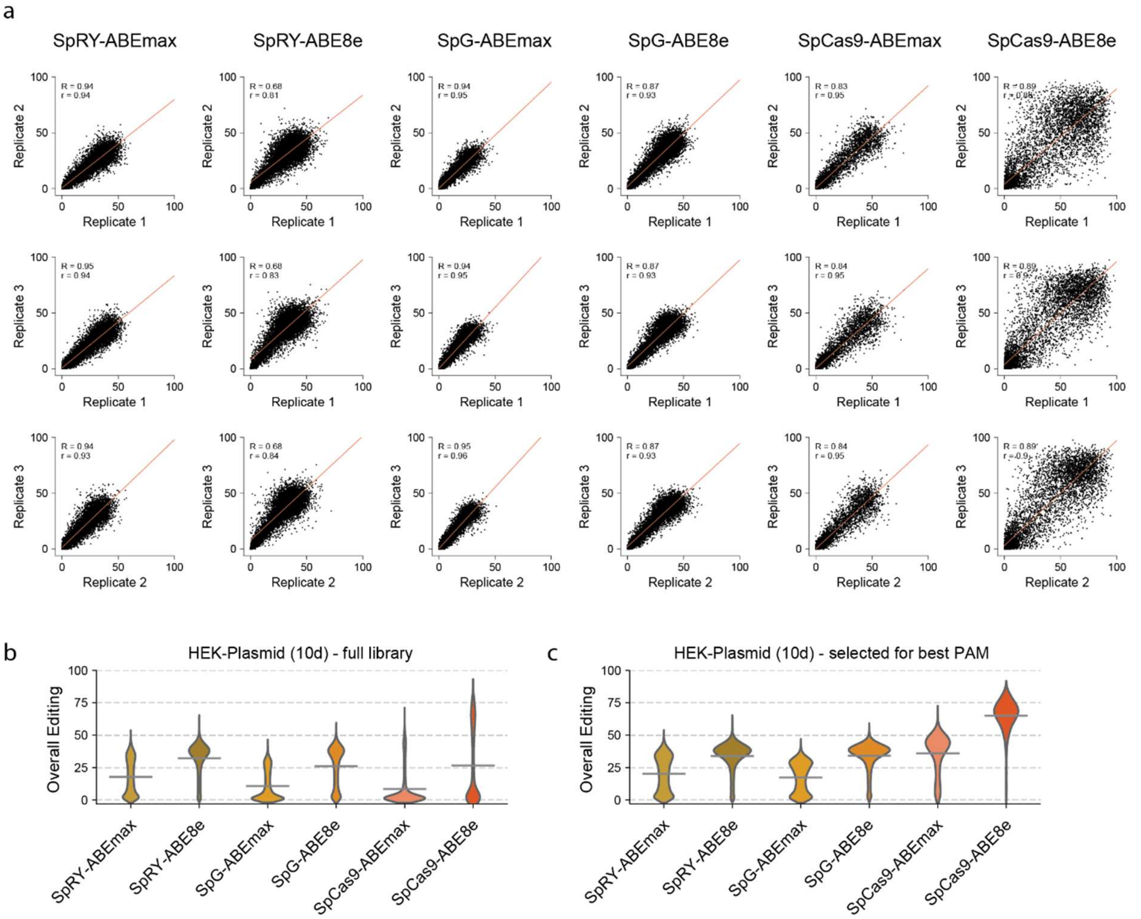

Fig. S2: Correlation of replicates of the HEK-Plasmid dataset with 10 days of ABE expression. a Scatter plots correlating editing efficiency of replicates treated with different base editors in HEK293T cells for 10 days (n for replicates of SpRY-ABEmax: 11497, SpRY-ABE8e: 11838, SpG-ABEmax: 9400, SpG-ABE8e: 10287, SpCas9-ABEmax: 9702, SpCas9-ABE8e: 7541). The red line represents linear regression. **b** Violin plots of base editor efficiencies with different ABE variants. n for SpRY-ABEmax: 11496, SpRY-ABE8e: 11838, SpG-ABEmax: 9400, SpG-ABE8e 10287, SpCas9-ABEmax: 9702, SpCas9-ABE8e: 7541. Mean editing efficiency is shown (grey line). **c** Violin plots of base editor efficiencies with different ABE variants. Datasets were filtered for optimal PAMs for the respective ABEs (NRN for SpRY, NGN for SpG and NGG for SpCas9). n for SpRY-ABEmax: 7644, SpRY-ABE8e: 7882, SpG-ABEmax: 3541, SpG-ABE8e 3786, SpCas9-ABEmax: 1030, SpCas9-ABE8e: 836.

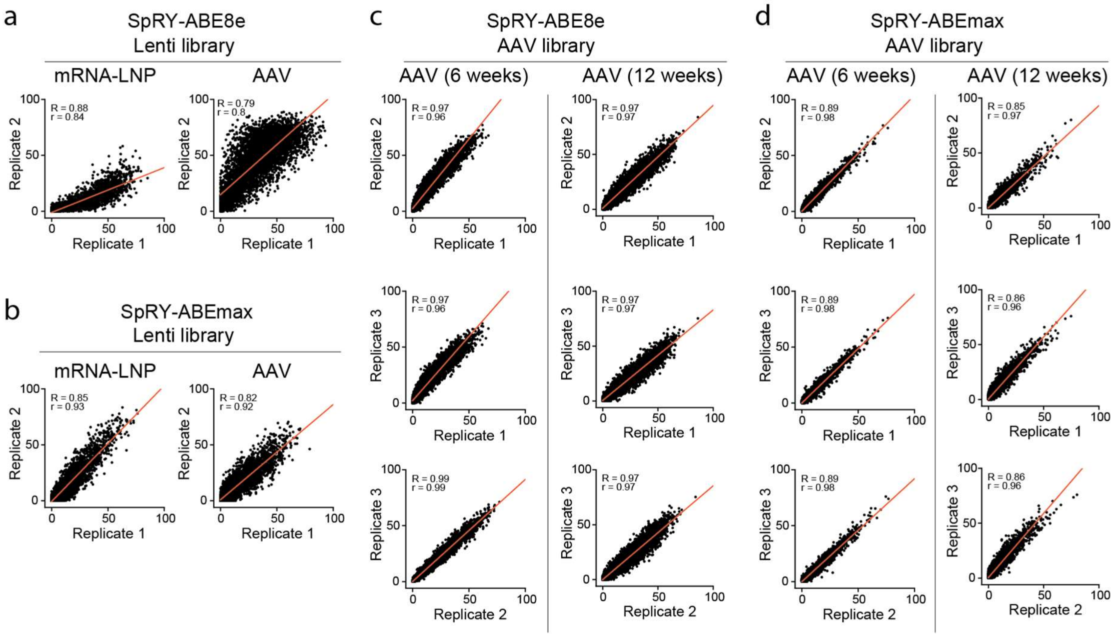

Fig. S3: Correlation of replicates for *in vivo* datasets. Scatter plots correlating editing efficiency of replicates of various *in vivo* datasets. The library was either delivered via lentivirus (**a,b**) or with AAV and sleeping beauty transposase (**c,d**). See methods section for more details. **a** SpRY-ABE8e delivered as mRNA-LNP (n = 2418) or AAV (n = 5233). **b** SpRY-ABEmax delivered as mRNA-LNP (n = 7818) or AAV (n = 8771). **c** SpRY-ABE8e delivered as AAV and analysis after 6 weeks (n = 8159) or 12 weeks (n = 9890). SpRY-ABEmax delivered as AAV and analysis after 6 weeks (n = 11446) or 12 weeks (n = 10657). The red line in all plots represents linear regression.

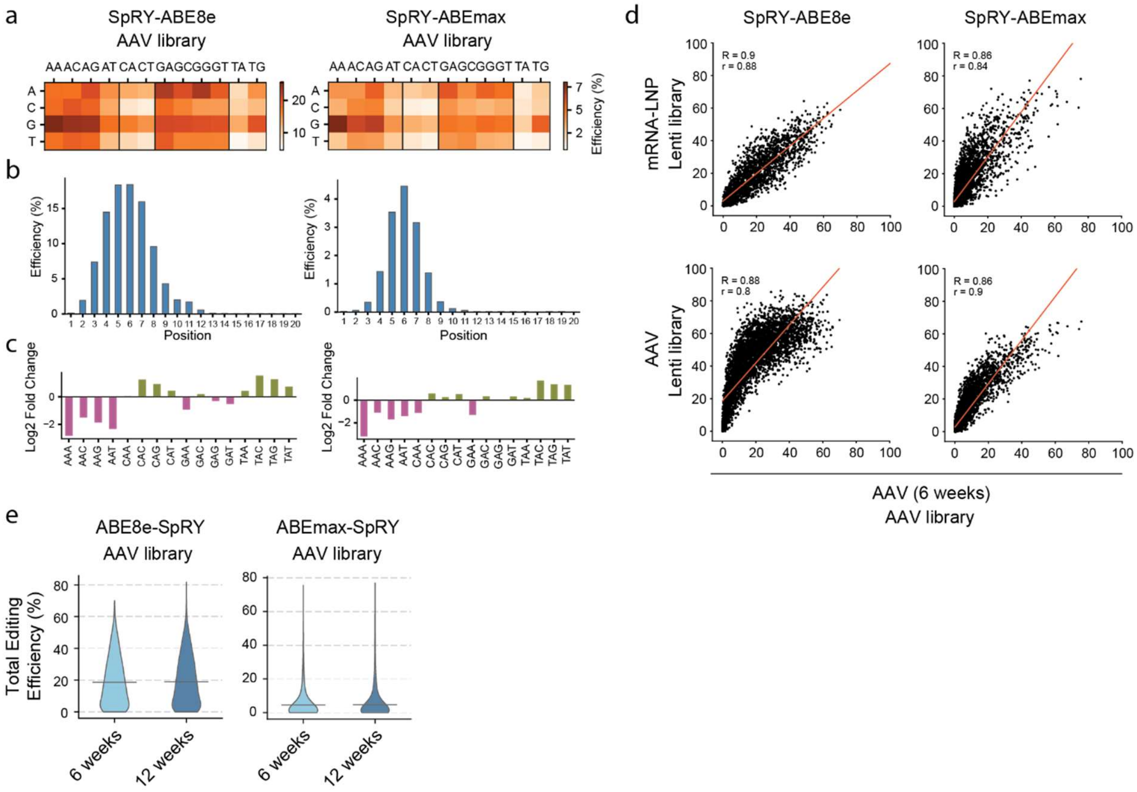

Fig. S4: Editing outcomes of the *in vivo* ABE screen with SB-integrated AAV libraries. **a-c** Screening results of AAV library delivered via sleeping beauty transposase. **a** Total editing efficiency for each PAM for SpRY-ABE8e (left) and SpRY-ABEmax (right). **b** Editing window for SpRY-ABE8e (left) and SpRY-ABEmax (right). Datasets were filtered for best PAMs (NRN). **c** Proportion of the different tri-nucleotide motifs for loci above mean editing efficiency (top) and below mean editing efficiency (bottom) for SpRY-ABE8e (left) and SpRY-ABEmax (right). **d** Scatter plot of correlation of various *in vivo* ABE datasets for SpRY-ABE8e (top, n =2418, 4919) and SpRY-ABEmax (bottom, n = 7812, 8766). **e** Violin plot of editing efficiency for AAV library delivered via sleeping beauty transposase for SpRY-ABE8e (left, n = 5434 and 6585) and SpRY-ABEmax (right, n = 7612 and 7081). Mean editing efficiency is given (grey line).

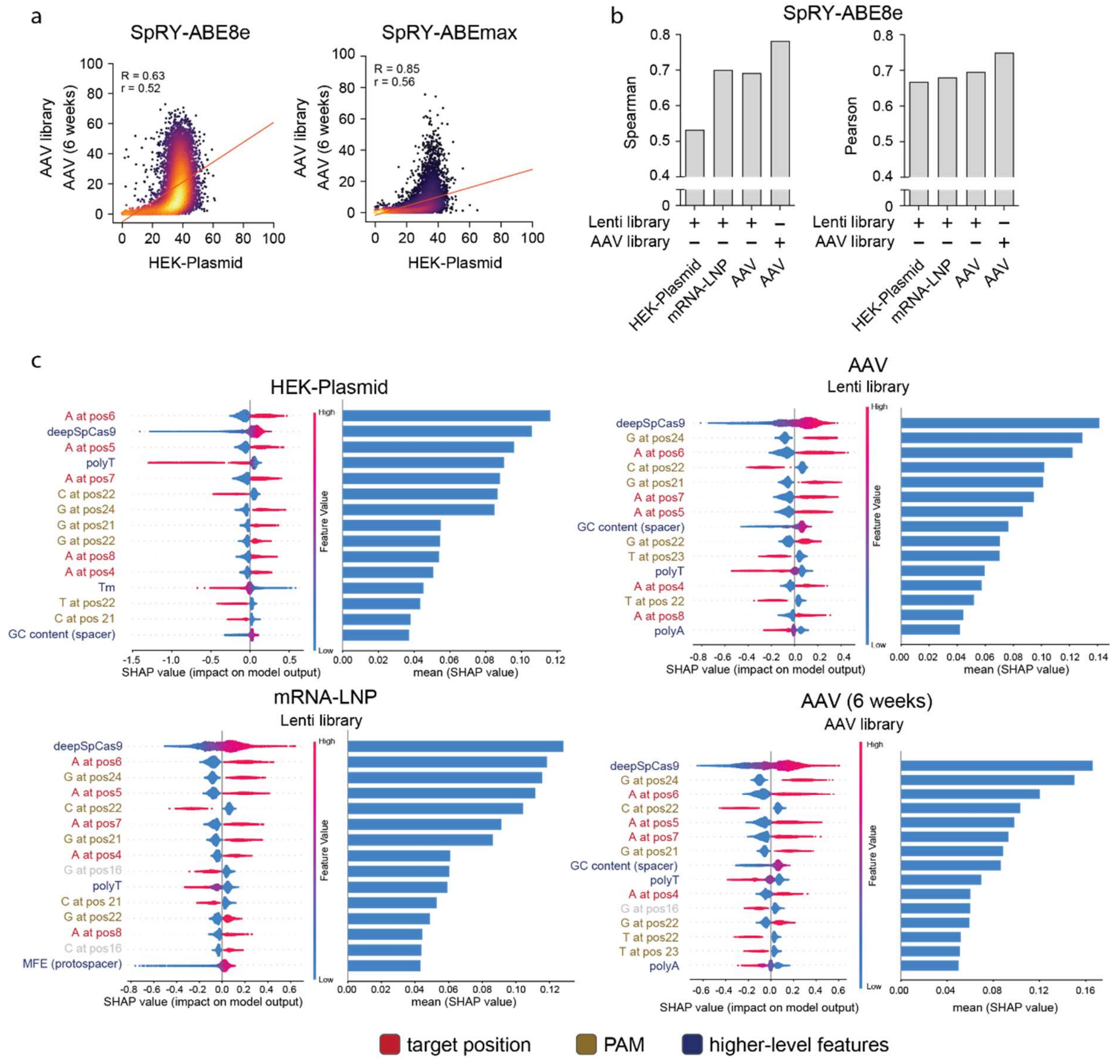

Fig. S5: Comparison of *in vitro* and *in vivo* ABE datasets. a Scatter plots correlating HEK-Plasmid and *in vivo* ABE screens with SB-integrated AAV library for SpRY-ABE8e (left, n = 8159) and SpRY-ABEmax (right, n = 11327). The red line represents linear regression. **b** Spearman and Pearson correlation of XGBoost Regression Model prediction accuracy on the corresponding test datasets. **c** SHAP analysis on XGBoost models. Top 15 features influencing the predicted outcome of editing efficiency are shown. High values or presence of feature are marked with red, and low values or absence of feature are marked with blue. Weight of SHAP values (visualized by barplots at the right of each feature) show feature importance in the prediction outcome. Features are grouped by colour in target position (dark red), PAM (dark yellow) and higher-level features (dark blue).

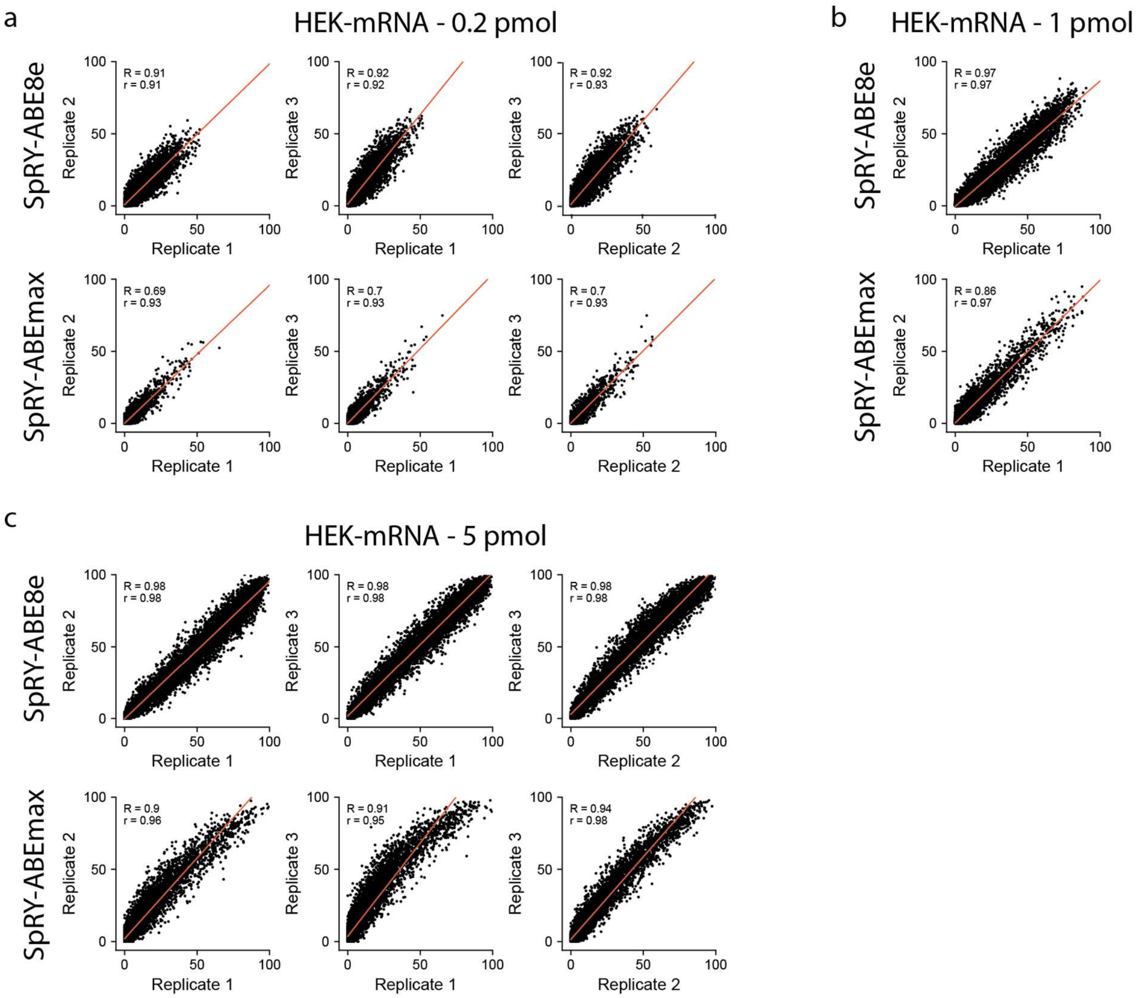

Fig. S6: Correlation of replicates in the HEK-mRNA dataset. a-c Scatter plot comparing editing efficiency of replicates of SpRY-ABE8e and SpRY-ABEmax for **a** 0.2 pmol ABE-mRNA (n for SpRY-ABE8e: 9580, SpRY-ABEmax: 9289) **b** 1 pmol ABE-mRNA (n for SpRY-ABE8e: 9697, SpRY-ABEmax: 9553) and **c** 5 pmol ABE-mRNA (n for SpRY-ABE8e: 8684, SpRY-ABEmax: 9018). The red line in all plots represents linear regression.

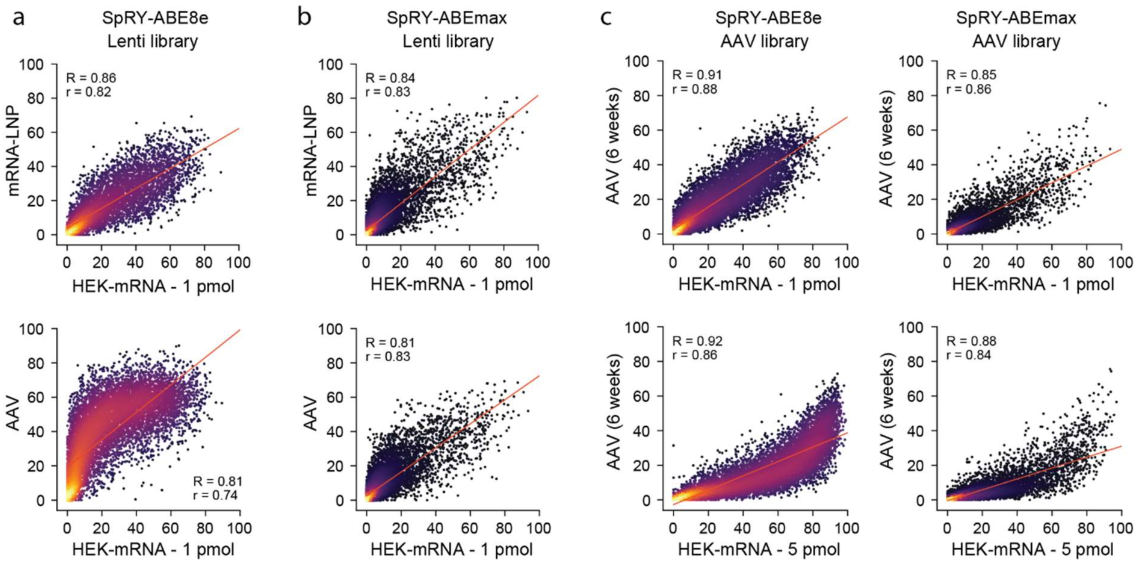

Fig. S7: Correlation of HEK-mRNA to *in vivo* datasets. a Scatter plots comparing editing efficiencies in HEK-mRNA (1pmol) and *in vivo* mRNA-LNP (top, n = 2405) or AAV (bottom, n = 5165) datasets for SpRY-ABE8e. **b** Scatter plots comparing editing efficiencies in HEK-mRNA (1pmol) and *in vivo* AAV mRNA-LNP (bottom, n = 7504) or AAV (bottom, n = 8188) datasets for SpRY-ABEmax. **c** Scatter plots comparing editing efficiencies in HEK-mRNA (1pmol and 5pmol) and *in vivo* AAV datasets generated with AAV library for SpRY-ABE8e (left, top n = 7824 and bottom n = 7399) and SpRY-ABEmax (right, top n = 9536 and bottom n = 9008). The red line represents linear regression.

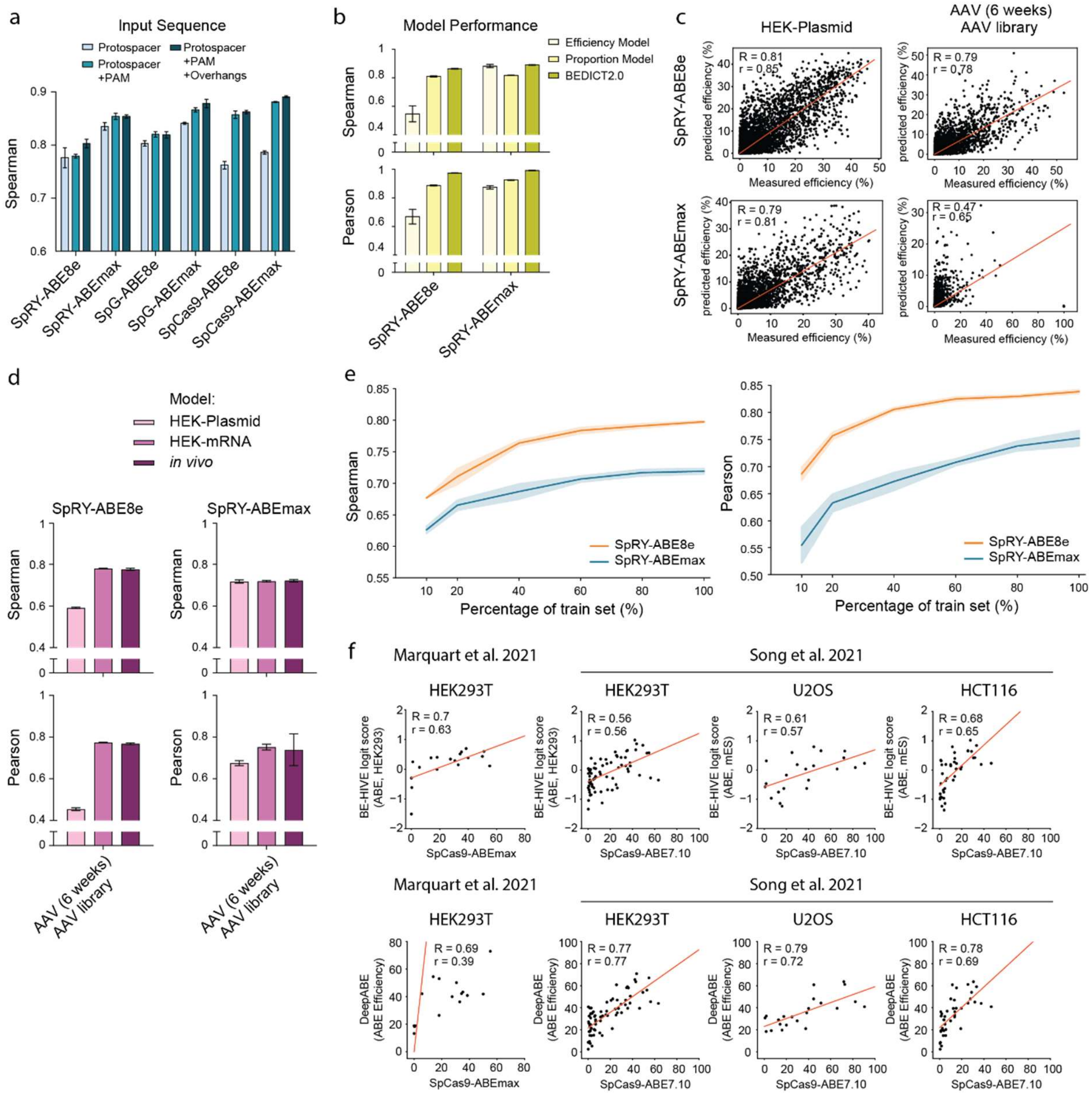

Fig. S8: Machine learning prediction of ABE activity by BEDICT2.0. a Performance of BEDICT1.2 on the same ABE dataset, but with different inputs for training. Models were trained on the following sequences: protospacer only, protospacer plus PAM, and protospacer plus PAM and overhangs. **b** Spearman and Pearson correlation for the prediction performance of the Efficiency Model, Proportion Model and BEDICT2.0. **c** Predicted efficiency by BEDICT2.0 plotted against measured efficiency of SpRY-ABE8e (top) and SpRY-ABEmax (bottom) in the Plasmid dataset and *in vivo* dataset of ABE screens with SB-integrated AAV library. Plots correspond to all edited variants (Proportion Model). The red line represents linear regression. **d** Comparison of Spearman and Pearson coefficients for BEDICT2.0 trained

on different datasets and tested on the *in vivo* dataset of ABE screens with SB-integrated AAV library. BEDICT2.0 was either trained on HEK-Plasmid dataset, HEK-mRNA dataset or the *in vivo* dataset directly. Datasets on the left were generated using SpRY-ABE8e, datasets on the right with SpRY-ABEmax. **e** Spearman and Pearson of prediction accuracy for ML models trained on mRNA SpRY-ABE8e and mRNA SpRY-ABEmax datasets. 100% represents the final BEDICT2.0 model. X-axis represents various split sizes of the training dataset (80% of the whole dataset). The validation and test sets (each 10% of the whole dataset) where kept constant. **f** Editing efficiency on endogenous loci compared to predictions of BE-HIVE (top row, [23]) and DeepABE (bottom row, [24]). n for Marquart-HEK293T [25]: 18, Song-HEK293T: 72, Song-U2OS: 22, Song-HCT116: 41 [24]. The red line represents linear regression.

Supplementary Notes

1 Detailed description of BEDICT version 2.0 model

Our model adapts and builds on the original BEDICT [1] model and introduces new architecture design changing the original an encoder-decoder architecture to an encoder-encoder one. This modification introduces two encoder blocks that independently encode both the target (wild type) and edited (outcome) DNA sequences into latent representations. This learned latent representation is then used to predict the probability of obtaining the output (i.e. edited) sequence from the target (wild type) DNA sequence as a result of applying a base editor. We refer to this modified version as the BEDICT1.2. It takes both the target DNA sequence and one of its corresponding outcome sequences and then returns the probability of obtaining such outcome sequence as a result of the base edit applied to the target DNA sequence.

Given the unique characteristics of the distribution across all possible outcomes for a given target sequence—having high mass on the wild type (i.e, no edit is observed) and relatively small values for other edited outcomes—we have designed a two-step modeling pipeline. It involves two distinct sub-models, namely the **Efficiency Model** and the **Proportion Model**. The Efficiency Model takes a target sequence as input and outputs the probability of the target sequence being edited (having non-wild type outcomes). The Proportion Model, on the other hand, takes the target sequence, presuming that all possible outcome sequences derived from that target sequence undergo editing, and outputs the probability of obtaining a given specific edited outcome (non-wild type). A comprehensive description of each sub-model is provided in the following sections.

1.1 Data representation

The selection of features to represent the input target sequence significantly influences the model's predictive accuracy. Initially, we explored various configurations such as using (a) only the protospacer, (b) adding PAM information, and (c) incorporating the surrounding contextual information such as overhangs. As mentioned in the main manuscript, including the overhangs in the model did not impact the model prediction performance. However, we found that an input sequence consisting of 20 bases protospacer followed by four bases of PAM information was the most optimal way to represent the target sequence.

Assume we have a target (reference, i.e. wild type) DNA sequence denoted as $\mathbf{x}_{\text{ref}} = [x_1, x_2, \ldots, x_T]$ where $x_i \in \{A, C, G, T\}$, and a set of DNA sequences $\mathbf{X}_{\text{out}} = [\mathbf{x}_{\text{out},1}, \mathbf{x}_{\text{out},2}, \ldots, \mathbf{x}_{\text{out},M}] \in \mathbb{R}^{M\times T}$ representing corresponding outcomes when a specific base editor is applied to the reference sequence $\mathbf{x}_{\text{ref}}$. The associated probabilities for these outcomes are given by $\mathbf{y} = [y_1, y_2, \ldots, y_M]$ where $y_i = P(\mathbf{x}_{\text{out},i}|\mathbf{x}_{\text{ref}}) \in [0,1]$, *for* $i = 1, 2, \ldots, M$, indicating the likelihood of obtaining outcome $\mathbf{x}_{\text{out},i}$ through editing of $\mathbf{x}_{\text{ref}}$. Here, T is the length of the reference sequence, and M represents the total number of possible outcomes for a given reference sequence. The number of outcome sequences can vary depending on the input reference sequence.

1.2 Two-step model

The probability of observing a specific outcome sequence $\mathbf{x}_{\text{out},i}$ for a given target/reference sequence $\mathbf{x}_{\text{ref}}$ after applying a certain base editor, can be described as $P(\mathbf{x}_{\text{out},i}|\mathbf{x}_{\text{ref}})$. Notably, the wild-type outcome often has a high probability, while various edited outcomes tend to have significantly lower probabilities of occurrence. Modeling $P(\mathbf{x}_{\text{out},i}|\mathbf{x}_{\text{ref}})$ directly proves challenging as the model struggles to discern rare outcomes with low probabilities. To address this, we propose a two-step model, decomposing $P(\mathbf{x}_{\text{out},i}|\mathbf{x}_{\text{ref}})$ into the product of two probabilities $P(\text{edited}|\mathbf{x}_{\text{ref}})$ and $P(\mathbf{x}_{\text{out},i}|\mathbf{x}_{\text{ref}}, \text{edited})$, from which we can also obtain $P(\mathbf{x}_{\text{out},i}|\mathbf{x}_{\text{ref}})$:

$$P(\mathbf{x}_{\text{out},i}|\mathbf{x}_{\text{ref}}) = \begin{cases} P(\mathbf{x}_{\text{out},i}|\mathbf{x}_{\text{ref}}, \text{edited})P(\text{edited}|\mathbf{x}_{\text{ref}}), & \text{if } \mathbf{x}_{\text{out},i} \neq \mathbf{x}_{\text{ref}} \\ 1 - P(\text{edited}|\mathbf{x}_{\text{ref}}), \text{if } \mathbf{x}_{\text{out},i} = \mathbf{x}_{\text{ref}} \end{cases} \tag{1}$$

With this new formulation, we learn two submodels. The first submodel is designed to learn the probability $P(\text{edited}|\mathbf{x}_{\text{ref}})$ which we refer to as the Efficiency Model. It operates by taking a target DNA sequence as input and computing the total efficiency score—a measure of the frequency of observing edited sequences at any position given a wild type sequence. The second submodel, referred to as the Proportion Model, is tailored to learn $P(\mathbf{x}_{\text{out},i}|\mathbf{x}_{\text{ref}}, \text{edited})P(\text{edited}|\mathbf{x}_{\text{ref}})$. This submodel takes both a target sequence and a specific edited outcome (non wild type) as inputs, generating the probability of observing that particular outcome sequence as a result of successful base editing on the target sequence.

1.2.1 Efficiency Model

The Efficiency Model focuses exclusively on the target sequence, overlooking the specific edit outcomes. Its main objective is to predict the probability of the target sequence undergoing modification, regardless of the nature of the edits. Hence, this submodel exclusively processes the input target sequence, which in our case is the concatenation of the protospacer and PAM, yielding the probability of the target sequence undergoing modification (resulting in non wild type outcomes). To achieve this, we propose to use a Convolutional Neural Network (CNN) on the one-hot encoding representation of the target sequence. More specifically, we use three layers of 1D-CNN (kernel size: 2, stride: 2) with filter sizes of 32, 64, and 128, respectively. Following the CNN layers, we apply a feed-forward network with ReLU activation, featuring a hidden layer dimension of 64. The output of this network is a two-dimensional vector, which is subsequently transformed into probabilities through the use of the Softmax function.

Objective function The Efficiency Model tackles a probabilistic classification task where the described CNN base neural network learns a function $f_{\theta_1}(\mathbf{x}_{\text{ref}})$ that parameterizes a binomial distribution $P(C|\mathbf{x}_{\text{ref}})$ of a random variable $C \in \{\textit{edited}, \textit{not edited}\}$ with the aim to learn to output the $P(C = \textit{edited}|\mathbf{x}_{\text{ref}})$ for a given reference sequence. To learn f_{θ_1}, we first computed the total editing efficiency for each reference sequence by summing all probabilities attributed to the non wild type outcomes by the following equation

$$P(\textit{edited}|\mathbf{x}_{\text{ref}}) = \frac{\textit{Sum of the read count of all edited reads for the target}}{\textit{Total read count of the target sequence}} \tag{2}$$

or equivalently $1 - P(\textit{wild-type}|\mathbf{x}_{ref})$. We trained f_{θ_1} using KL-divergence loss that is applied on the true distribution $P(C|\mathbf{x}_{\text{ref}})$ and learned distribution $\hat{P}_{\theta_1}(C|\mathbf{x}_{\text{ref}})$ for each reference sequence.

$$\mathcal{L}_{\textit{efficiency}}(\theta_1, D) = \sum_{i=1}^{N} D_{kl}(P(C|\mathbf{x}^i_{\text{ref}}) \| \hat{P}_{\theta_1}(C|\mathbf{x}^i_{\text{ref}})) \tag{3}$$

1.2.2 Proportion Model

The Proportion Model focuses on predicting the probability of the different edited outcomes (i.e. edited sequences) for a given target sequence (wild type sequence), i.e., $P(X_{\text{out}}|\mathbf{x}_{\text{ref}}, \textit{edited})$. Therefore, this submodel takes both the target sequence as well as one of its corresponding outcome sequences and outputs the probability of observing such an outcome. To learn this distribution, we first process the data by removing the wild-type from each reference sequence's corresponding output X_{out}, then normalizing the probabilities of the remaining outcomes to ensure a valid distribution.By this process, we effectively converting $P(X_{\text{out}}|\mathbf{x}_{\text{ref}})$ into the distribution $P(X_{\text{out}}|\mathbf{x}_{\text{ref}}, \textit{edited})$.

We learn a function $f_{\theta_2}(\mathbf{x}_{\text{ref}}, \mathbf{x}_{\text{out},i})$ which parameterizes the distribution $P(X_{\text{out}}|\mathbf{x}_{\text{ref}}, \textit{edited})$. We learn $f_{\theta_2}(\mathbf{x}_{\text{ref}}, \mathbf{x}_{\text{out},i})$ using two encoder networks, and one prediction network. The architectures of the two encoding networks, one for the target sequence and the other for the outcome sequence are identical, however, they do not share any parameter. Consequently, we will only describe in detail one of these networks in the following sections. The target/reference sequence encoder network comprises of two components: an **embedding layer** and an **encoder block**.

Embedding Layer The embedding layer embeds both the nucleotides and their corresponding position (in the protospacer) from the one-hot encoded representation to a dense vector representation. Given a protospacer sequence extended with its corresponding PAM site: $\mathbf{x}_{\text{ref}} = [x_1, x_2, \ldots, x_T] \in \mathbb{R}^T$, x_t represents the nucleotide at position t. In our case, T=24. We use $\mathbf{O} = [\mathbf{o}_1, \mathbf{o}_2, \ldots, \mathbf{o}_T] \in \mathbb{R}^{K \times T}$ as the corresponding one-hot encoded representation. As there are four distinct nucleotides (A, T, C and G) we set $K = 4$. An embedding matrix $\mathbf{W}_e$ is used to map each $\mathbf{o}_t \in \mathbb{R}^K$ to a fixed-length vector representation:

$$\mathbf{e}_t = \mathbf{W}_e \mathbf{o}_t \tag{4}$$

where $\mathbf{W}_e \in \mathbb{R}^{d_e \times K}$, $\mathbf{e}_t \in \mathbb{R}^{d_e}$, and d_e is the embedding dimension we chose.
Similarly, each nucleotide's position in the sequence $\mathbf{x}_{\text{ref}}$ is represented by one-hot encoding with a dictionary size of T. An embedding matrix $\mathbf{W}_{p'} \in \mathbb{R}^{d_e \times T}$ is applied to project the $\mathbf{p}_t$ to a dense vector representation:

$$\mathbf{p}'_t = \mathbf{W}_{p'} \mathbf{p}_t \tag{5}$$

where $\mathbf{W}_{p'} \in \mathbb{R}^{d_e \times T}$, $\mathbf{p}'_t \in \mathbb{R}^{d_e}$. Both embeddings $\mathbf{e}_t$ and $\mathbf{p}'_t$ are summed to get a unified representation for every element x_t in the reference sequence $\mathbf{x}_{\text{ref}}$.

$$\mathbf{u}_t = \mathbf{e}_t + \mathbf{p}'_t \quad \forall\ t = 1, 2, \ldots T \tag{6}$$

This results in the embedded representation $\mathbf{U} = [\mathbf{u}_1, \mathbf{u}_2, \ldots, \mathbf{u}_T]$ of the reference sequence.

Encoder Block To learn a good representation that takes into account the relationships between the nucleotides in the reference sequence, we use a multi-head self-attention to encode the embedded representation. Multi-head Attention is a module that employs multiple single-head self-attention in parallel (i.e. simultaneously) for processing each input vector $\mathbf{u}_t$. The outputs from every single-head layer are then concatenated and transformed by an affine transformation to generate a fixed-length vector.

The single-head self-attention approach [2] learns three different linear projections of the input vector using three separate matrices: (1) a queries matrix $\mathbf{W}_{query}$, (2) a keys matrix $\mathbf{W}_{key}$, and (3) a values matrix $\mathbf{W}_{value}$. Each input $\mathbf{u}_t$ in $\mathbf{U}$ is mapped using these matrices to compute three new vectors:

$$\mathbf{q}_t = \mathbf{W}_{query}\mathbf{u}_t \tag{7}$$

$$\mathbf{k}_t = \mathbf{W}_{key}\mathbf{u}_t \tag{8}$$

$$\mathbf{v}_t = \mathbf{W}_{value}\mathbf{u}_t \tag{9}$$

(10)

where $\mathbf{W}_{query}$, $\mathbf{W}_{key}$, $\mathbf{W}_{value} \in \mathbb{R}^{d \times d_e}$, $\mathbf{q}_t, \mathbf{k}_t, \mathbf{v}_t \in \mathbb{R}^d$ are query, key and value vectors, and d is the dimension of the those projected vectors. In the second step, attention scores are computed using the pairwise similarity between the query and key vectors for each position t in the sequence. The similarity is defined by first computing a scaled dot product between the pairwise vectors and then normalizing it using the softmax function. At each position t, we compute attention scores α_{tl} representing the similarity between t-th query $\mathbf{q}_t$ and l-th key $\mathbf{k}_l$.

$$score(\mathbf{q}_t, \mathbf{k}_l) = \frac{\mathbf{q}_t^T \mathbf{k}_l}{\sqrt{d}} \tag{11}$$

$$\alpha_{tl} = \frac{\exp(score(\mathbf{q}_t, \mathbf{k}_l))}{\sum_{l=1}^{T} \exp(score(\mathbf{q}_t, \mathbf{k}_l))} \tag{12}$$

Then a weighted sum of value vector $\mathbf{v}_l$ using attention α_{tl}, $\forall\, l \in \{1, 2, \ldots, T\}$ is performed to generate a new vector representation $\mathbf{e}_t \in \mathbb{R}^d$ at position t.

$$\mathbf{e}_t = \sum_{l=1}^{T} \alpha_{tl}\mathbf{v}_l \tag{13}$$

This process is applied to every position in the original embedding of the sequence, $\mathbf{U}$, to obtain a sequence of vectors $\mathbf{E} = [\mathbf{e}_1, \mathbf{e}_2, \ldots, \mathbf{e}_T]$.

In a multi-head setting with H number of heads, the queries, keys, and values matrices will be indexed by superscript h (i.e. $\mathbf{W}^h_{query}$, $\mathbf{W}^h_{key}$, $\mathbf{W}^h_{value} \in \mathbb{R}^{d \times d_e}$) and applied separately to generate a new vector representation $\mathbf{e}^h_t$ for every singe-head self-attention layer. The output from each single-head attention layer is concatenated into one vector $\mathbf{e}^{concat}_t = concat(\mathbf{e}^1_t, \mathbf{e}^2_t, \ldots, \mathbf{e}^H_t)$ where $\mathbf{e}^{concat}_t \in \mathbb{R}^{dH}$. Then it goes through an affine transformation using $\mathbf{W} \in \mathbb{R}^{d \times dH}$ and $\mathbf{b} \in \mathbb{R}^d$ to generate the encoded representation $\hat{\mathbf{Z}} = [\hat{\mathbf{z}}_1, \hat{\mathbf{z}}_2, \ldots, \hat{\mathbf{z}}_T]$ of the reference sequence

$$\hat{\mathbf{z}}_t = \mathbf{W}\mathbf{e}^{concat}_t + \mathbf{b} \tag{14}$$

To improve the gradient flow in layers during training, we additionally use residual connections / skip-connections [3]. This is done by summing both the newly computed output of the current layer with the output from the previous layer. In our setting, a first residual connection sums the output of the self-attention layer $\hat{\mathbf{z}}_t$ and the output of embedding block $\mathbf{u}_t$ for each position t in the sequence.

We also deploy layer normalization [4] after the self-attention layer with the goal of ameliorating the "covariate-shift" problem by re-standardizing the computed vector representations (i.e. using the mean and variance across the features/embedding dimension d). Given a computed vector $\hat{\mathbf{z}}_t$, the LayerNorm function will standardize the input vector using the mean and variance along the dimension of the feature d and apply scaling and shifting steps. After the layer normalization, the learned representation goes through a feed-forward network with one hidden layer and ReLu activation function. Subsequently, residual connection followed by layer normalization a is applied to the output of this feed-forward network to obtain the learned representation $\mathbf{z}_t \in \mathbb{R}^d$. Eventually, the encoder block transformed the embedded vectors in sequence $\mathbf{U}$ to the learned representation $\mathbf{Z} = [\mathbf{z}_1, \mathbf{z}_2, \ldots, \mathbf{z}_T] \in \mathbb{R}^{T \times d}$ that incorporates the contextual information/relationships between features through attention.

The above process describes one encoding block. We stack N encoding blocks to construct our encoder network. In the Proportion Model, we apply two encoder networks with the same network architecture on the reference sequence and corresponding outcome sequence. This yields two encoded representations which we

denote by $\mathbf{Z}^{ref} \in \mathbb{R}^{T\times d}$ and $\mathbf{Z}^{out} \in \mathbb{R}^{T\times d}$ respectively. We then concatenated them in the feature dimension to generate one common representation $\mathbf{Z} = [\mathbf{z}_1, \mathbf{z}_2, \ldots, \mathbf{z}_T] \in \mathbb{R}^{T\times 2d}$.

Output network The output network $g(\mathbf{Z})$ takes as input the final representation $\mathbf{Z} \in \mathbb{R}^{T\times 2d}$ and performs an affine transformation followed by softmax operation to compute the probability of edit of every target base (i.e. base A or C depending on the chosen base editor) as it is shown below:

$$\hat{y}_{it} = \sigma(\mathbf{W}\mathbf{z}_{it} + \mathbf{b}_t) \tag{15}$$

where $\mathbf{W} \in \mathbb{R}^{2\times 2d}, \mathbf{b}_t \in \mathbb{R}^2$ and σ is a softmax function. $\hat{y}_{it}$ represents the probability of editing occurring at the t-th position in the i-th outcome sequence. The un-normalized probability for the whole i-th output sequence $\mathbf{x}_{\text{out},i}$ given its reference sequence is computed by $\hat{y}_i = \prod_{t=1}^{T} \hat{y}_{i,t}$, which is then normalized across all the outcomes to make a valid probability distribution (Eq. 16). Therefore, the approximated probability for obtaining i-th edited (non-wild type) outcome sequence is given by:

$$\hat{P}(\mathbf{x}_{\text{out},i} \mid \mathbf{x}_{\text{ref}}, \textit{edited}) = \frac{\hat{y}_i}{\sum_{i=1}^{M} \hat{y}_i} \tag{16}$$

Note that, we use the same length of input and outcome sequence. However, our input sequence also includes PAM information, and editing only happens in the protospacer. Moreover, due to the nature of the Base editor, only specific nucleotides, in our case adenine (A), get edited while other nucleotides are not affected. Therefore, we use a masking technique to only consider the positions that are possible to be edited and mask out other positions. Therefore, the PAM information (or the contextual information such as left/right overhangs is participating by affecting the embedding of the nucleotides in the protospacer but is not considered in the loss as they are not changed/edited.

Objective Function We used the Kullback–Leibler (KL) divergence on the model's estimated distribution over all outcome sequences for a given reference sequence $\mathbf{x}^i_{\text{ref}}$ and the actual distribution:

$$D^i_{\text{KL}}(P(X_{\text{out}}|\mathbf{x}^i_{\text{ref}}, \textit{edited}) || \hat{P}(X_{\text{out}}|\mathbf{x}^i_{\text{ref}}, \textit{edited})) = \sum_{j=1}^{M_i} P(\mathbf{x}_{\text{out},j}|\mathbf{x}^i_{\text{ref}}, \textit{edited}) \log \frac{P(\mathbf{x}_{\text{out},j}|\mathbf{x}^i_{\text{ref}}, \textit{edited})}{\hat{P}(\mathbf{x}_{\text{out},j}|\mathbf{x}^i_{\text{ref}}, \textit{edited})}$$

Lastly, the objective function for the whole training set is defined by the average loss across all the reference sequences as follows:

$$\mathcal{L}_{\text{proportion}}(\theta_{\mathbf{2}}; D) = \sum_{i=1}^{N} D^i_{KL}(P(X_{\text{out}} \mid \mathbf{x}^i_{\text{ref}}, \textit{edited}) || \hat{P}_{\theta_2}(X_{\text{out}} \mid \mathbf{x}^i_{\text{ref}}, \textit{edited})$$

1.3 Objective function

The final objective is composed of both the Efficiency Model loss and the Proportion Model loss with a weight regularization term (i.e. l_2-norm regularization) applied to the model parameters represented by $\theta = \{\theta_{\mathbf{1}}, \theta_{\mathbf{2}}\}$ (Eq. 17)

$$\mathcal{L}(\theta; D) = \mathcal{L}_{\textit{efficiency}}(\theta_{\mathbf{1}}; D) + \mathcal{L}_{\textit{proprotion}}(\theta_{\mathbf{2}}, D) + \frac{\lambda}{2}\|\theta\|_2^2 \tag{17}$$

where D denotes the training data, consisting of pairs of target sequences along with their corresponding output sequences, each accompanied by its respective probability. It's important to note that the two objectives have distinct sets of parameters. As a result, we conducted separate training sessions for the Efficiency Model and the Proportion Model.

1.4 Hyperparameters

The diagram below presents two lists: List 1 and List 2, detailing the hyperparameter values explored in our experiments.

List 1 Efficiency Model hyperparameters option

- Convolutional layers
  - 1st 1D convolutional layer
    - Input channels .... $\{4\}$
    - Output channels .... $\{32\}$
    - Kernel size .... $\{2\}$
  - 2nd 1D convolutional layer
    - Input channels .... $\{32\}$
    - Output channels .... $\{64\}$
    - Kernel size .... $\{2\}$
  - 3rd 1D convolutional layer
    - Input channels .... $\{64\}$
    - Output channels .... $\{128\}$
    - Kernel size .... $\{2\}$
  - Non-linear function .... $\{ReLU\}$
- MLP layer
  - hidden layer dimension .... $\{64\}$
  - Non-linear function .... $\{ReLU\}$
- l_2-norm regularization λ .... $\{10^{-2}\}$
- Batch size during training $|B|$ .... $\{100\}$
- Optimization algorithm .... $\{Adam\}$
- Maximum epochs .... $\{300\}$

List 2 Proportion Model hyperparameters option

- Embedding Block operations
  - Nucleotide embedding layer
    - embedding dimension d' .... $\{128\}$
  - Position embedding layer
    - embedding dimension d' .... $\{128\}$
- Encoder Block operations
  - Self-attention layer
    - Number of attention heads .... $\{8\}$
    - Attention type .... $\{Narrow\}$
  - Dropout .... $\{0.25\}$
  - Feed-Forward layer
    - MLP embedding factor (multiplier) ξ .... $\{2\}$
    - Non-linear function .... $\{ReLU\}$
  - Number of repeats for Encoder Block .... $\{1\}$
- l_2-norm regularization λ .... $\{10^{-4}\}$
- Batch size during training $|B|$ .... $\{400\}$
- Optimization algorithm .... $\{Adam\}$

2 Comparative Analysis of Model Performance Against Established Literature Baselines

We compared our model with BE-DICT [1] which is one of the relevant existing models that tackle base editing outcome prediction. BE-DICT is a sequence-to-sequence model where the decoding happens in an auto-regressive manner, it is computationally heavy compared to our proposed method. Moreover, it uses only the protospacer to represent the target sequence. We extended and retrained BE-DICT on two of the datasets (randomly chosen) and compared the prediction results with ours. For a fair comparison, we first used our one-step model trained using only the protospacer as target sequence representation. The results of this experiment (Table 1) reveal the advantages of the architectural changes, particularly in adopting an encoder-encoder architecture over the traditional sequence-to-sequence (encoder-decoder) model.

		BE-DICT		BEDICT1.2	
reference sequence	Libraries	Spearman	Pearson	Spearman	Pearson
prrotospacer	SpRY-ABEmax	0.801	0.943	0.835	0.981
	SpRY-ABE8e	0.746	0.861	0.776	0.965

Table 1: Performance comparison with the baselines

Then we extended their model to use both the protospacer and PAM to represent the reference sequence and compared it with our proposed two-step model which is also trained using protospacer and PAM information as the target sequence.

		BE-DICT		BEDICT2.0	
reference sequence	Libraries	Spearman	Pearson	Spearman	Pearson
prrotospacer & PAM	SpRY-ABEmax	0.804	0.951	0.873	0.986
	SpRY-ABE8e	0.762	0.850	0.862	0.974

Table 2: Performance comparison with the baselines

Results in Table 2 show that our model consistently outperforms BE-DICT. Furthermore, considering computational efficiency during model training (on SpRY-ABE8e data using the protospacer and PAM reference sequence representation), BE-DICT takes in the order of minute per epoch (wall time), while our model accomplishes the same task in the order of seconds (15 seconds).

This highlights the benefits of avoiding the complex sequence-to-sequence architecture in favor of a streamlined encoder-encoder structure. This choice not only improves the computational efficiency but also leads to model predictive performance improvements. Moreover, the introduction of a two-stage model and usage of PAM together with the protospacer as target sequence representation amplifies these performance gains even further.

To assess our model's performance against other state-of-the-art models, we conducted evaluations using the test sets provided by these models. Table 3 displays our findings, which include three most recent models: BE-HIVE [5], DeepABE [6], and BEDICT [1], along with their respective test sets labeled as A. et al., S. et al., and M. et al.

	All Outocmes			Non wild-types		
Datasets	A.et all	S. et al	M. et al	A.et all	S. et al	M. et al
BEDICT	0.96	0.94	0.86	0.81	0.90	0.82
DeepABE	0.87	0.93	0.84	0.86	0.96	0.84
BE-HIVE	0.71	0.88	0.74	0.92	0.93	0.81
Our model	0.972	0.974	0.972	0.939	0.945	0.953

Table 3: Model performance on the test set from the different published studies. Columns represent test sets, rows represent models used

The idea is to take the published trained model and evaluate their performance on those various test sets. For the three baseline models, we refer to the results reported in the BEDICT paper. As for our model, to ensure fairness in comparison, we used our single-step model trained on SpG-ABEmax libraries since most baselines,

except DeepABE, do not incorporate the PAM as input. Note that BEDICT 2.0 takes input as the protospacer and PAM information to represent the input sequence. By using our BEDICT1.2 we make sure the comparison is fair, moreover, in Table 3 and 2 we have shown that BEDICT2.0 have superior performance over BEDICT1.2.

The results correspond to two scenarios: 1) considering all possible outcomes, and 2) only considering non wild type outcomes. The results for the non wild type outcomes correspond to the model prediction where we only consider non wild outcomes. In the case of non wild type outcome prediction, we mention that other models were trained exclusively on non-wild outcomes, with outcomes per sequence being renormalized. Our one-stage model, however, was trained on data encompassing all outcomes, so we report non-wild-type results with outcomes renormalized for a fair comparison.

Finally, we also tested the prediction accuracy of our model with the prediction accuracy of the most recent model called DeepBE [7]. We have trained our model on one of the datasets they provide (SpCas9-NG-ABE8e(V106W)) and compared our result with the ones reported in their paper. This data includes 7112 unique target sequences and overall 28261 input-output pairs. The data they provide is processed such that only bases from position 3 to 10 (editing window size of 8) are considered to be edited. The data has train/test separation where 6340 among 7112 target sequences are selected as training and the rest as a test set. We trained our model on their training set and tested our model performance on their test set (Table 4).

Model	Pearson correlation	Spearman correlation
DeepBE [7]	0.83	0.91
Ours	0.89	0.96

Table 4: Comparing with DeepBE performance on one of their dataset corresponding to SpCas9-NG-ABE8e(V106W) base editor

3 References

[1] K. F. Marquart, A. Allam, S. Janjuha, A. Sintsova, L. Villiger, N. Frey, M. Krauthammer, and G. Schwank, "Predicting base editing outcomes with an attention-based deep learning algorithm trained on high-throughput target library screens," *Nature Communications*, vol. 12, no. 1, p. 5114, 2021.

[2] A. Vaswani, N. Shazeer, N. Parmar, J. Uszkoreit, L. Jones, A. N. Gomez, Ł. Kaiser, and I. Polosukhin, "Attention is all you need," *Advances in neural information processing systems*, vol. 30, 2017.

[3] K. He, X. Zhang, S. Ren, and J. Sun, "Deep residual learning for image recognition," in *Proceedings of the IEEE conference on computer vision and pattern recognition*, 2016, pp. 770–778.

[4] J. L. Ba, J. R. Kiros, and G. E. Hinton, "Layer normalization," *arXiv preprint arXiv:1607.06450*, 2016.

[5] M. Arbab, M. W. Shen, B. Mok, C. Wilson, Ż. Matuszek, C. A. Cassa, and D. R. Liu, "Determinants of base editing outcomes from target library analysis and machine learning," *Cell*, vol. 182, no. 2, pp. 463–480, 2020.

[6] M. Song, H. K. Kim, S. Lee, Y. Kim, S.-Y. Seo, J. Park, J. W. Choi, H. Jang, J. H. Shin, S. Min *et al.*, "Sequence-specific prediction of the efficiencies of adenine and cytosine base editors," *Nature biotechnology*, vol. 38, no. 9, pp. 1037–1043, 2020.

[7] N. Kim, S. Choi, S. Kim, M. Song, J. H. Seo, S. Min, J. Park, S.-R. Cho, and H. H. Kim, "Deep learning models to predict the editing efficiencies and outcomes of diverse base editors," *Nature Biotechnology*, pp. 1–14, 2023.
